# Duration of effectiveness of vaccines against SARS-CoV-2 infection and COVID-19 disease: results of a systematic review and meta-regression

**DOI:** 10.1016/S0140-6736(22)00152-0

**Published:** 2022-03-05

**Authors:** Daniel R Feikin, Melissa M Higdon, Laith J Abu-Raddad, Nick Andrews, Rafael Araos, Yair Goldberg, Michelle J Groome, Amit Huppert, Katherine L O'Brien, Peter G Smith, Annelies Wilder-Smith, Scott Zeger, Maria Deloria Knoll, Minal K Patel

**Affiliations:** a Department of Immunisations, Vaccines, and Biologicals, WHO, Geneva, Switzerland; b International Vaccine Access Center, Department of International Health, John Hopkins Bloomberg School of Public Health, Baltimore, MA, USA; c Department of Epidemiology, John Hopkins Bloomberg School of Public Health, Baltimore, MA, USA; d Infectious Disease Epidemiology Group, Weill Cornell Medicine–Qatar, Doha, Qatar; e UK Health Security Agency, London, UK; f Instituto de Ciencias e Innovacion en Medicina, Facultad de Medicina, Clinica Alemana Universidad del Desarrollo, Santiago, Chile; g Advanced Centre for Chronic Diseases, Santiago, Chile; h Technion Israel Institute of Technology, Haife, Israel; i The Gertner Institute for Epidemiology and Health Policy Research, Sheba Medical Centre, Tel Aviv University, Tel Aviv, Israel; j National Institute for Communicable Diseases, Division of the National Health Laboratory Service, Johannesburg, South Africa; k School of Pathology, Faculty of Health Sciences, University of the Witwatersrand, Johannesburg, South Africa; l MRC International Statistics and Epidemiology Group, London School of Hygiene & Tropical Medicine, London, UK

## Abstract

**Background:**

Knowing whether COVID-19 vaccine effectiveness wanes is crucial for informing vaccine policy, such as the need for and timing of booster doses. We aimed to systematically review the evidence for the duration of protection of COVID-19 vaccines against various clinical outcomes, and to assess changes in the rates of breakthrough infection caused by the delta variant with increasing time since vaccination.

**Methods:**

This study was designed as a systematic review and meta-regression. We did a systematic review of preprint and peer-reviewed published article databases from June 17, 2021, to Dec 2, 2021. Randomised controlled trials of COVID-19 vaccine efficacy and observational studies of COVID-19 vaccine effectiveness were eligible. Studies with vaccine efficacy or effectiveness estimates at discrete time intervals of people who had received full vaccination and that met predefined screening criteria underwent full-text review. We used random-effects meta-regression to estimate the average change in vaccine efficacy or effectiveness 1–6 months after full vaccination.

**Findings:**

Of 13 744 studies screened, 310 underwent full-text review, and 18 studies were included (all studies were carried out before the omicron variant began to circulate widely). Risk of bias, established using the risk of bias 2 tool for randomised controlled trials or the risk of bias in non-randomised studies of interventions tool was low for three studies, moderate for eight studies, and serious for seven studies. We included 78 vaccine-specific vaccine efficacy or effectiveness evaluations (Pfizer–BioNTech-Comirnaty, n=38; Moderna-mRNA-1273, n=23; Janssen-Ad26.COV2.S, n=9; and AstraZeneca-Vaxzevria, n=8). On average, vaccine efficacy or effectiveness against SARS-CoV-2 infection decreased from 1 month to 6 months after full vaccination by 21·0 percentage points (95% CI 13·9–29·8) among people of all ages and 20·7 percentage points (10·2–36·6) among older people (as defined by each study, who were at least 50 years old). For symptomatic COVID-19 disease, vaccine efficacy or effectiveness decreased by 24·9 percentage points (95% CI 13·4–41·6) in people of all ages and 32·0 percentage points (11·0–69·0) in older people. For severe COVID-19 disease, vaccine efficacy or effectiveness decreased by 10·0 percentage points (95% CI 6·1–15·4) in people of all ages and 9·5 percentage points (5·7–14·6) in older people. Most (81%) vaccine efficacy or effectiveness estimates against severe disease remained greater than 70% over time.

**Interpretation:**

COVID-19 vaccine efficacy or effectiveness against severe disease remained high, although it did decrease somewhat by 6 months after full vaccination. By contrast, vaccine efficacy or effectiveness against infection and symptomatic disease decreased approximately 20–30 percentage points by 6 months. The decrease in vaccine efficacy or effectiveness is likely caused by, at least in part, waning immunity, although an effect of bias cannot be ruled out. Evaluating vaccine efficacy or effectiveness beyond 6 months will be crucial for updating COVID-19 vaccine policy.

**Funding:**

Coalition for Epidemic Preparedness Innovations.

## Introduction

Almost 2 years into the COVID-19 pandemic, several COVID-19 vaccines have received Emergency Use Listing or Emergency Use Authorisation (EUL or EUA) by regulatory authorities and WHO on the basis of vaccine efficacy results from randomised controlled trials.^1^ Efficacy results at the time of EUL or EUA, however, had a median follow-up time after full vaccination of only 2–3 months. Estimates of vaccine effectiveness among people vaccin-ated as part of national vaccine rollouts were similar to the efficacy results in the first few months after vaccine introduction.^2^ Assessing the duration of protection for COVID-19 vaccines over longer time periods, however, requires continued monitoring. Knowing whether and to what extent vaccine effectiveness wanes is crucial to inform vaccine policy decisions, such as the need for, timing, and target populations for booster doses.

**Research in context**

**Evidence before this study**

Approximately 1 year after the first introductions of COVID-19 vaccines, many studies have been published that assess vaccine efficacy and effectiveness after full vaccination. Several systematic reviews of studies on COVID-19 vaccine efficacy and effectiveness have been published, but none focused on how vaccine efficacy or effectiveness changes with time since vaccination. We systematically reviewed the evidence for changes in COVID-19 vaccine efficacy or effectiveness with time since full vaccination for various clinical outcomes. Additionally, our review summarises evidence for rates of breakthrough infections caused by the delta variant among people who were vaccinated, stratified by time since vaccination. In interpreting these studies, we discuss potential biases in evaluating changes in vaccine effectiveness with time since vaccination. We searched for studies that evaluated vaccine efficacy or effectiveness at discrete time intervals after full vaccination from June 17, 2021 to Dec 2, 2021 in PubMed, Embase, medRxiv, bioRxiv, khub, Research Square, SSRN, Eurosurveillance.org, Europepmc.org, and the WHO COVID-19 database, which compiles searches of more than 100 databases, including Scopus, Web of Science, and grey literature. We searched for studies with several variations of the primary key search terms “COVID-19”, “SARS-CoV-2”, and “vaccine” (including names of specific vaccines) and “randomized controlled trial” or “vaccine effectiveness” (including names of specific study designs). We also searched regulatory agency databases. Studies were included if they presented vaccine efficacy or effectiveness estimates at discrete time intervals from people who were fully vaccinated compared with those who were unvaccinated for SARS-CoV-2 infection, COVID-19 symptomatic disease, or severe disease, for any vaccine that has received Emergency Use Listing by WHO. Vaccine efficacy or effectiveness estimates confined to a single variant were analysed separately from those obtained from a mixture of variants. Random-effects meta-regression was used to estimate the mean change in vaccine efficacy or effectiveness from 1 month to 6 months after full vaccination. After applying exclusion criteria, we included 18 studies of vaccine efficacy or effectiveness at discrete time intervals after full vaccination and seven studies in which risk of breakthrough infection could be assessed by time of vaccination. In addition, the same search strategy was used to find studies presenting analyses of breakthrough infections, in which the rate, risk, or odds of COVID-19 outcomes among different vaccine cohorts (ie, vaccinated at different times) were included.

**Added value of this study**

We found that during the 6 months after full vaccination, vaccine efficacy or effectiveness against SARS-CoV-2 infection and symptomatic COVID-19 disease decreased by approximately 20–30 percentage points, on average, for the four vaccines that we evaluated. By contrast, most studies showed that vaccine efficacy or effectiveness against severe disease was maintained above 70% after full vaccination, with minimal decrease to 6 months (approximately 9–10 percentage points). This is the first systematic review and meta-regression to date, to our knowledge, that describes the timing and magnitude of decreasing vaccine efficacy or effectiveness over time since full vaccination, by disease outcome.

**Implications of all the available evidence**

Studies of the duration of protection of COVID-19 vaccine effectiveness indicate that vaccine effectiveness decreases more against infection and symptomatic disease than against severe disease in the 6 months after full vaccination. This decreasing vaccine efficacy or effectiveness is probably caused by, at least in part, waning immunity. Several biases, however, can affect estimates of declining vaccine efficacy or effectiveness over time. Whether vaccine efficacy or effectiveness will eventually decrease further against severe disease, and in the setting of new variants such as omicron, requires ongoing evaluation at later timepoints after full vaccination. Policy makers considering the need and timing of booster doses should integrate vaccine-specific and outcome-specific evidence of decreasing vaccine efficacy or effectiveness with other considerations, such as vaccine coverage and supply, prioritisation relative to primary-series vaccination, programmatic issues, and local COVID-19 epidemiology.

Several systematic reviews of COVID-19 efficacy and effectiveness studies have been published, but none have evaluated the duration of protection of COVID-19 vaccines.^3^, ^4^, ^5^, ^6^, ^7^, ^8^ We systematically reviewed the evidence for the duration of protection of COVID-19 vaccines against various clinical outcomes by assessing studies that evaluate vaccine efficacy or effectiveness at various time periods after vaccination. Additionally, we established rates of breakthrough infection due to the delta variant among vaccinated people stratified by time since vaccination.

## Methods

### Search strategy and selection criteria

Since June 17, 2021, WHO and the International Vaccine Access Center at Johns Hopkins Bloomberg School of Public Health (MA, USA) have been tracking the emerging evidence for COVID-19 vaccine efficacy or effectiveness and have posted their methodology and updated weekly results on the VIEW-HUB website.^9^ For this systematic review, we followed PRISMA guidelines (appendix pp 2–5) and considered peer-reviewed and preprint studies published from June 17, 2021, to Dec 2, 2021. Randomised controlled trials of COVID-19 vaccine efficacy and observational studies of COVID-19 vaccine effectiveness were eligible. We searched the following databases and preprint servers without language restrictions: PubMed, Embase, medRxiv, BioRxiv, khub, Research Square, SSRN, Eurosurveillance.org, Europepmc.org, and the WHO COVID-19 database, which compiles searches of more than 100 databases, including Scopus, Web of Science, and grey literature. The search strategy is described in the appendix (p 6). During full-text review, a vaccine efficacy or effectiveness study was excluded if it did not meet predefined criteria (appendix p 7). Only vaccine efficacy or effectiveness estimates that compared people who were fully vaccinated with those who were unvaccinated were included; we excluded estimates that included people who were partially vaccinated. In addition, we searched the US Food and Drug Administration and European Medicines Agency websites for manufacturer applications for approval of additional or booster doses. Discrepancies in study inclusion were resolved by discussion among three investigators (MMH, MDK, and MKP).

Most COVID-19 vaccine efficacy or effectiveness studies have given results as cumulative vaccine efficacy or effectiveness after full vaccination through variable time periods of follow-up. However, cumulative vaccine efficacy or effectiveness estimates over several months can distort estimates of waning immunity, particularly if most cases occur in the earlier or later parts of the follow-up period. Therefore, we applied a second set of inclusion and exclusion criteria after the initial search, undertaken by two investigators (MKP and MMH). First, studies were included if they presented several vaccine efficacy or effectiveness estimates for discrete time intervals after the final dose in the primary series. Second, to allow sufficient time for potential waning to occur, studies were excluded if they did not provide at least one vaccine efficacy or effectiveness estimate 3 months after the final dose (appendix pp 8–9). Third, we excluded studies that combined several vaccines in vaccine effectiveness estimates because vaccines of differing effectiveness were often introduced at different times to varying target populations, which could lead to confounding of vaccine effectiveness estimates at different time intervals.

An approach to disaggregate decreasing vaccine effectiveness caused by waning immunity from decreased effectiveness due to a newly prevalent variant is to compare rates or risks of vaccine breakthrough infections by time since vaccination during a time period in which a single variant is predominant. For this approach, we considered studies of breakthrough infection (ie, infection or disease among people who were fully vaccinated only) identified through the full-text review. One study was eligible for both analyses.^10^ We included studies that provided risk ratios, rate ratios, or odds ratios of breakthrough infection (or provided data to calculate them) among different vaccine-recipient cohorts (ie, people vaccinated at different times). We only included studies that identified cases during periods in which delta was the predominant variant.

All studies that met the inclusion criteria for both analyses were evaluated for bias using the risk of bias 2 tool for randomised controlled trials or the risk of bias in non-randomised studies of interventions tool.^11^, ^12^

### Data analysis

Populations, intervention, comparators, and outcomes are described (appendix p 10). For the main analysis, the primary outcome measure was vaccine efficacy or effectiveness and 95% CI at each time interval after the final dose of the primary vaccine series. We extracted adjusted vaccine efficacy or effectiveness results for each outcome (infection, symptomatic disease, and severe disease) by vaccine, age group (all ages and older people), and variant setting. We only extracted vaccine efficacy or effectiveness estimates for time intervals during which a person could have been fully vaccinated considered as having received the complete primary vaccine schedule followed by enough time to develop immunological protection, as defined in the clinical trials for each vaccine (ie, ≥7 days from the second dose for Pfizer–BioNTech Comirnaty and ≥14 days from the second dose for AstraZeneca–Vaxzevria and Moderna-mRNA-1273 and from the first dose of Janssen-Ad26.COV2.S). Because vaccine efficacy or effectiveness might have been lower against some variants of concern (VOCs) and the prevalence of VOCs in a study population could change over time,^4^ we evaluated vaccine efficacy or effectiveness estimates for two variant settings separately. In the first variant setting, we evaluated vaccine efficacy or effective-ness estimates over time for a single VOC, either as determined by genomic sequencing or during a period when that variant was predominant, including from settings with only non-VOC variants, and from settings with both non-VOCs and Alpha variants, because of minimal differences in vaccine efficacy or effectiveness.^13^ In the second setting, we evaluated settings in which there was a mixture of variants over time, including some periods with non-Alpha VOCs in circulation. To visually show the duration of vaccine efficacy or effectiveness over time, we plotted vaccine efficacy or effectiveness at the median timepoint for each time interval separately by outcome, age group, and variant context (appendix pp 11–12). The set of vaccine efficacy or effectiveness estimates over time for each unique study-vaccine grouping are shown.

The average change in vaccine efficacy or effectiveness over time was estimated using a linear mixed-effects model for the repeated measures within each study-vaccine group (PROC MIXED, SAS version 9.4; appendix pp 13–14). We regressed the log of 1 minus vaccine efficacy or effectiveness on the log of months since vaccination (to maintain a linear relationship between vaccine efficacy or effectiveness and time in months). SEs of the natural logarithm of 1 minus vaccine efficacy or effectiveness, derived from the 95% CIs for the vaccine efficacy or effectiveness reported by each study, were squared to produce estimates of residual variances for inverse weighting in the linear mixed-effects model. The model had a random intercept and slope over time for each study-vaccine group (ie, each line in figures). For vaccine efficacy or effectiveness estimates of 100% for which 95% CIs were not estimable, we approximated the SEs using study data and added 0·5 cases to each group. We excluded vaccine efficacy or effectiveness estimates with 95% CIs for which the lower bound was up to 0% and the upper bound was 100%, because 95% CIs were uninformative. Models were run for each outcome, age group, and variant context combination. Because we did not observe substantial differences in the results for single-variant versus mixed-variant settings, we also estimated the change in vaccine efficacy or effectiveness combining both variant settings to increase precision around summary estimates.

For the analysis of vaccine breakthrough deltainfections, we extracted data on study design, population size, testing period, vaccines in use, age group, outcome, cases, and denominator for cohorts of people grouped by time since final dose. We calculated incidence rates or risk from case and denominator data for each vaccinated cohort. Incidence rate or risk ratios (IRRs) were calculated by dividing the incidence rate or risk of each vaccinated cohort by that of a reference group. The vaccinated cohort most recently vaccinated was used as the reference group. 95% CIs for IRRs were calculated from raw study data using the Byar method for rates and the Taylor series method for risks.^14^, ^15^ Studies presenting adjusted odds ratios of breakthrough infection with 95% CIs were also included (n=3).^16^, ^17^, ^18^ Incidence rate or risk, and odds ratios with 95% CIs were visualised on graphs for each vaccinated cohort.

### Role of the funding source

The Coalition for Epidemic Preparedness Innovations (CEPI) supports the ongoing literature review and data abstraction. CEPI had no role in the study design, data analysis, data interpretation, or writing of this report.

## Results

**Figure 1:**
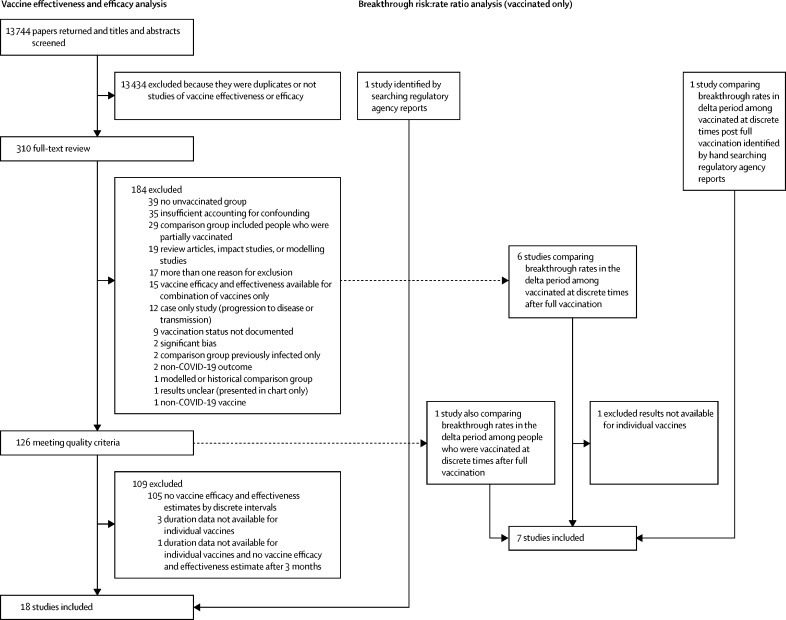
Study selection

13 744 studies were screened, and 310 underwent full-text review (figure 1). After applying two sets of inclusion and exclusion criteria, 18 studies were included in the vaccine efficacy or effectiveness analysis. Seven studies were peer-reviewed publications, ten were not peer-reviewed (eg, preprints or Morbidity and Mortality Weekly Report publications), and one study came from a regulatory application. Three studies were randomised controlled trials^19^, ^20^, ^21^ and 15 were post-introduction observational studies (seven were test-negative design case-control studies, six were retrospective studies, and two were prospective cohort studies; table 1).^10^, ^22^, ^23^, ^24^, ^25^, ^26^, ^27^, ^28^, ^29^, ^30^, ^31^, ^32^, ^33^, ^34^, ^35^ Studies were done in Canada (one study), Finland (one study), Israel (one study), Qatar (one study), Spain (one study), Sweden (one study), the UK (two studies), the USA (eight studies), and in addition two multicountry clinical trials were carried out. The Canadian study included separate results for Quebec and British Columbia; therefore, the results for each province were considered separately for this review.^31^ Among these 18 studies, there were 78 vaccine-specific vaccine efficacy or effectiveness evaluations (Pfizer–BioNTech-Comirnaty, n=38; Moderna-mRNA-1273, n=23; Janssen-Ad26.COV2.S, n=9; and AstraZeneca-Vaxzevria, n=8).

**Table 1:** Characteristics and results of included vaccine efficacy and effectiveness studies

	**Study design (variables controlled for in the vaccine efficacy and effectiveness estimates)**	**Vaccine**	**Age group**	Disease outcome*	**Variant to which vaccine efficacy or effectiveness estimates apply**†	**Time interval since final dose, days**	**Vaccine efficacy or effectiveness (95% CI)**
Goldberg et al (Israel)^10^	Retrospective cohort (age, week of infection, past PCR tests, sex, and demographic group)	Pfizer–BioNTech (Comirnaty)	≥60 years	Any infection	Delta‡	41–91§	82 (70 to 89)
						72–121§	81 (73 to 86)
						102–137§	73 (67 to 79)
						118–152§	74 (68 to 79)
						133–166§	67 (59 to 73)
						147–180§	63 (58 to 67)
						161–196§	57 (52 to 62)
			≥60 years	Severe disease	Delta‡	102–152§	92 (87 to 95)
						133–180§	88 (84 to 91)
						161–211§	85 (81 to 88)
El Sahly et al (USA)^19^	Randomised controlled trial	Moderna (mRNA-1273)	≥18 years	Any symptomatic disease	Mixture of variants	7–59	91·8 (86·9 to 95·1)
						2–119	94·0 (91·2 to 96·1)
						≥120	92·4 (84·3 to 96·8)
Thomas et al (several countries)^20^	Randomised controlled trial	Pfizer–BioNTech (Comirnaty)	≥12 years	Any symptomatic disease	Mixture of variants	7–59	96·2 (93·3 to 98·1)
						60–119	90·1 (86·6 to 92·9)
						≥120	83·7 (74·7 to 89·9)
FDA (several countries)^21^	Randomised controlled trial	Janssen (Ad26.COV2.S)	≥18 years	Any symptomatic disease	Mixture of variants	15–28	72·3 (62·1 to 80·1)
						29–56	61·7 (52·5 to 69·2)
						57–112	50·8 (40·2 to 59·7)
						≥113	45·2 (33·0 to 55·3)
			≥18 years	Severe disease	Mixture of variants	15–28	65·5 (27·3 to 85·0)
						29–56	85·7 (71·0 to 93·7)
						57–112	67·8 (44·2 to 82·2)
						≥113	71·7 (51·4 to 84·3)
Chemaitelly et al (Qatar)^22^	Test negative case-control study (sex, age group, nationality, reason for PCR testing, and calendar week of PCR test)	Pfizer–BioNTech (Comirnaty)	≥12 years	Any infection	Delta	31–60	73·3 (63·6 to 80·4)
						61–90	62·4 (50·2 to 71·6)
						91–120	35·1 (14·7 to 50·6)
						121–150	20·4 (−1·9 to 37·8)
						151–214	17·9 (−12·9 to 40·3)
			≥60 years	Any infection	Mixture of variants	31–60	71·9 (65·4 to 77·2)
						61–90	67·4 (57·4 to 75·1)
						91–120	53·3 (26·9 to 70·2)
						121–150	79·3 (50·2 to 91·4)
						151–180	15·4 (−88·8 to 62·1)
						>180	6·6 (−93·4 to 54·9)
			≥12 years	Severe disease	Mixture of variants	31–60	96·8 (93·9 to 98·3)
						61–90	94·3 (89·1 to 97·0)
						91–120	83·7 (65·5 to 92·3)
						121–150	100 (75·5 to 100)¶
						151–180	88·9 (52·1 to 97·4)
						>180	55·6 (−44·3 to 86·3)
			≥60 years	Severe disease	Mixture of variants	31–60	96·5 (90·4 to 98·7)
						61–90	90·4 (79·2 to 95·6)
						91–120	78·3 (42·8 to 91·7)
						121–150	100·0 (31·6 to 100)¶
						151–180	66·7 (−220·5 to 96·5)
						>180	50·0 (−451·4 to 95·5)
Martinez-Baz et al (Spain)^23^	Prospective cohort (age, sex, chronic conditions, contact setting, month, and vaccination status of index case)	Janssen (Ad26.COV2.S)	≥18 years	Any infection	Mixture of variants	<90	52 (44 to 59)
						≥90	28 (−8 to 53)
		Moderna (mRNA-1273)	≥18 years	Any infection	Mixture of variants	<90	85 (80 to 88)
						≥90	67 (50 to 78)
		Pfizer–BioNTech (Comirnaty)	≥18 years	Any infection	Mixture of variants	<90	70 (67 to 73)
						≥90	63 (58 to 68)
Thompson et al (USA)^24^	Test negative case-control study (age, geographical region, calendar time, local virus circulation, and propensity for vaccination)	Moderna (mRNA-1273)	≥50 years	Severe disease	Alpha and non-variant of concern*	28–41	89 (83 to 93)
						42–55	93 (87 to 97)
						56–69	96 (92 to 98)
						70–83	86 (75 to 92)
						84–97	93 (82 to 97)
						>111	95 (79 to 99)
		Pfizer–BioNTech (Comirnaty)	≥50 years	Severe disease	Alpha and non-variant of concern‡	28–41	95 (91 to 97)
						42–55	86 (79 to 91)
						56–69	83 (75 to 89)
						70–83	90 (82 to 94)
						84–97	87 (76 to 93)
						98–111	75 (57 to 85)
						>111	83 (64 to 92)
						28–41	89 (83 to 93)
Andrews et al (UK)^25^	Test negative case-control study (age, sex, deprivation index, ethnic group, care home residence, geographical region, calendar week, health and social care worker status, and clinical risk group or a clinically vulnerable group)	Vaxzevria (AstraZeneca)	≥16 years	Any symptomatic disease	Delta	14–69	66·7 (66·3 to 67·0)
						70–104	59·3 (58·8 to 59·9)
						105–139	52·6 (51·7 to 53·5)
						>139	47·3 (45·0 to 49·6)
			≥65 years	Any symptomatic disease	Delta	14–69	58·9 (54·8 to 62·6)
						70–104	49·9 (45·4 to 54·0)
						105–139	43·3 (38·1 to 48·0)
						>139	36·6 (28·7 to 43·7)
			≥16 years	Severe disease	Delta	14–69	95·2 (94·6 to 95·6)
						70–104	91·4 (90·5 to 92·2)
						105–139	86·8 (85·1 to 88·4)
						>139	77·0 (70·3 to 82·3)
			≥65 years	Severe disease	Delta	14–69	92·2 (89·4 to 94·3)
						70–104	90·2 (87·8 to 92·2)
						105–139	85·4 (81·6 to 88·5)
						>139	76·3 (65·3 to 83·8)
		Pfizer–BioNTech (Comirnaty)	≥16 years	Any symptomatic disease	Delta	14–69	89·8 (89·6 to 90·0)
						70–104	80·3 (79·9 to 80·6)
						105–139	73·4 (72·9 to 73·9)
						>139	69·7 (68·7 to 70·5)
			≥65 years	Any symptomatic disease	Delta	14–69	80·1 (77·5 to 82·4)
						70–104	69·1 (66·2 to 71·8)
						105–139	62·1 (58·6 to 65·4)
						>139	55·3 (50·2 to 60·0)
			≥16 years	Severe disease	Delta	14–69	98·4 (97·9 to 98·8)
						70–104	96·5 (95·9 to 97·1)
						105–139	94·4 (93·4 to 95·2)
						>139	92·7 (90·3 to 94·6)
			≥65 years	Severe disease	Delta	14–69	97·9 (95·9 to 99·0)
						70–104	95·7 (94·3 to 96·8)
						105–139	93·0 (90·9 to 94·6)
						>139	90·7 (86·0 to 93·8)
Bruxvoort et al (USA)^26^	Test negative case-control study (age, sex, race or ethnicity, specimen collection date, smoking, comorbidities, frailty index, pregnancy, history of COVID-19, number of outpatient visits, catchment area, and specimen type)	Moderna (mRNA-1273)	≥18 years	Any infection	Delta	14–60	94·1 (90·5 to 96·3)
						61–90	88·7 (85·0 to 91·5)
						91–120	85·9 (81·1 to 89·5)
						121–150	77·0 (69·1 to 82·9)
						151–180	80·0 (70·2 to 86·6)
			≥65 years	Any infection	Delta	14–60	52·9 (0·0 to 86·6)
						61–90	85·7 (57·9 to 95·1)
						91–120	85·8 (68·9 to 93·5)
						121–150	62·3 (32·4 to 79·0)
						151–180	90·8 (25·6 to 98·9)
Self et al (USA)^27^	Test negative case-control study (age, sex, race or ethnicity, admission date, and region)	Moderna (mRNA-1273)	≥18 years	Severe disease	Mixture of variants	14–120	93 (90 to 95)
						>120	92 (87 to 96)
		Pfizer–BioNTech (Comirnaty)	≥18 years	Severe disease	Mixture of variants	14–120	91 (88 to 93)
						>120	77 (67 to 84)
Tartof et al (USA)^28^	Retrospective cohort (age, sex, race, ethnicity, previous PCR positive test, previous health-care use, comorbidities, influenza and pneumococcal vaccination status, and deprivation index)	Pfizer–BioNTech (Comirnaty)	≥16 years	Any infection	Delta	37–66	88 (81 to 92)
						67–96	78 (70 to 83)
						97–126	60 (48 to 69)
						>126	53 (39 to 65)
			≥65 years	Any infection	Mixture of variants	37–66	79 (70 to 85)
						67–96	75 (65 to 83)
						97–126	56 (45 to 65)
						127–156	49 (41 to 57)
						>156	43 (30 to 54)
			≥16 years	Severe disease	Mixture of variants	37–66	89 (84 to 92)
						67–96	92 (89 to 95)
						97–126	93 (89 to 95)
						127–156	91 (87 to 93)
						>156	88 (82 to 92)
			≥65 years	Severe disease	Mixture of variants	37–66	88 (78 to 93)
						67–96	89 (78 to 94)
						97–126	86 (77 to 92)
						127–156	85 (77 to 90)
						>156	83 (69 to 90)
Lin et al (USA)^29^	Retrospective cohort (age, sex, race or ethnicity, geographical region, and county-level vaccination rate)	Janssen (Ad26.COV2.S)	≥12 years	Any symptomatic disease	Mixture of variants	31–60	71·4 (68·3 to 74·2)
						61–90	71·1 (68·2 to 73·6)
						91–120	61·8 (59·3 to 64·1)
						121–150	59·4 (57·2 to 64·5)
						151–180	64·0 (60·3 to 67·4)
			≥65 years	Any symptomatic disease	Mixture of variants	31–60	73·1 (61·6 to 81·2)
						61–90	63·4 (50·7 to 72·9)
						91–120	51·9 (40·7 to 61·0)
						121–150	44·5 (34·4 to 53·1)
						151–180	43·3 (25·6 to 56·8)
			≥12 years	Severe disease	Mixture of variants	31–60	88·6 (76·4 to 94·5)
						61–90	89·0 (76·0 to 94·9)
						91–120	78·5 (63·6 to 87·3)
						121–150	88·1 (78·3 to 93·5)
						151–180	51·7 (−19·7 to 80·5)
			≥65 years	Severe disease	Mixture of variants	31–60	82·9 (49·3 to 94·3)
						61–90	89·4 (61·3 to 97·1)
						91–120	64·9 (26·1 to 83·4)
						121–150	78·4 (53·2 to 90·0)
						151–180	4·6 (−175·5 to 66·9)
		Moderna (mRNA-1273)	≥12 years	Any symptomatic disease	Mixture of variants	31–60	92·5 (91·9 to 93·1)
						61–90	91·5 (90·9 to 92·0)
						91–120	87·6 (87·1 to 88·2)
						121–150	83·4 (82·7 to 84·1)
						151–180	80·3 (79·3 to 81·2)
						181–210	77·8 (75·9 to 79·6)
			≥65 years	Any symptomatic disease	Mixture of variants	31–60	90·2 (88·5 to 91·6)
						61–90	89·8 (88·1 to 91·3)
						91–120	83·0 (81·2 to 84·7)
						121–150	79·5 (78·0 to 80·8)
						151–180	75·4 (73·8 to 77·0)
						181–210	67·0 (62·6 to 70·8)
			≥12 years and ≥65 years	Severe disease	Mixture of variants	31–60	94·5 (92·0 to 96·3)
						61–90	96·4 (94·6 to 97·6)
						91–120	94·5 (92·4 to 96·0)
						121–150	93·2 (91·1 to 94·8)
						151–180	91·4 (88·4 to 93·6)
						181–210	91·8 (83·4 to 95·9)
				Severe disease	Mixture of variants	31–60	91·6 (87·2 to 94·5)
						61–90	95·0 (91·8 to 97·0)
						91–120	91·4 (87·5 to 94·0)
						121–150	90·0 (86·8 to 92·4)
						151–180	89·6 (85·7 to 92·5)
						181–210	87·6 (73·9 to 94·1)
Lin et al (USA)^29^	Retrospective cohort (age, sex, race or ethnicity, geographical region, and county-level vaccination rate)	Pfizer–BioNTech (Comirnaty)	≥12 years	Any symptomatic disease	Mixture of variants	7–36	94·5 (94·1 to 94·9)
						37–66	88·2 (87·5 to 88·8)
						67–96	84·1 (83·4 to 84·7)
						97–126	80·4 (79·8 to 81·0)
						127–156	75·9 (75·1 to 76·7)
						157–186	66·6 (65·2 to 67·8)
						187–216	67·8 (65·9 to 69·7)
			≥65 years	Any symptomatic disease	Mixture of variants	7–36	92·7 (91·5 to 93·8)
						37–66	87·6 (85·6 to 89·2)
						67–96	85·2 (83·2 to 87·0)
						97–126	74·3 (72·0 to 76·4)
						127–156	66·7 (64·6 to 68·6)
						157–186	57·4 (55·0 to 59·7)
						187–216	60·1 (55·3 to 64·4)
			≥12 years	Severe disease	Overall	7–36	96·2 (94·4 to 97·4)
						37–66	93·8 (91·1 to 95·6)
						67–96	95·2 (93·2 to 96·6)
						97–126	91·4 (88·9 to 93·4)
						127–156	89·5 (86·6 to 91·7)
						157–186	86·6 (82·9 to 89·5)
						187–216	88·4 (80·0 to 93·2)
			≥65 years	Severe disease	Overall	7–36	95·7 (93·2 to 97·3)
						37–66	90·2 (85·5 to 93·4)
						67–96	91·0 (86·7 to 94·0)
						97–126	85·1 (79·8 to 89·0)
						127–156	86·5 (82·4 to 89·6)
						157–186	82·1 (76·7 to 86·2)
						187–216	81·4 (65·1 to 90·1)
Nordstrom et al (Sweden)^30^	Retrospective cohort (age, sex, date of second dose, homemaker service, place of birth, education, and comorbidities)	Vaxzevria (AstraZeneca)	≥16 years	Any symptomatic disease	Mixture of variants	31–60	49 (28 to 64)
						61–120	41 (29 to 51)
						>120	19 (−97 to 28)
		Moderna (mRNA-1273)	≥16 years	Any symptomatic disease	Mixture of variants	31–60	93 (90 to 94)
						61–120	85 (82 to 88)
						121–180	71 (56 to 81)
						>180	59 (18 to 79)
		Pfizer–BioNTech (Comirnaty)	≥16 years	Any symptomatic disease	Mixture of variants	31–60	89 (88 to 90)
						61–120	85 (84 to 85)
						121–180	47 (39 to 55)
						181–210	29 (15 to 42)
						>210	23 (−2 to 41)
Skowronski et al (Canada)^31^	Test negative case-control study (age, sex, week of analysis period, and region)	Moderna (mRNA-1273)	≥18 years	Any infection	Delta	28–55	92 (91 to 93)
						56–83	91 (89 to 92)
						84–111	88 (86 to 90)
						112–139	87 (81 to 91)
						140–167	91 (81 to 95)
						>167	85 (61 to 95)
			≥70 years	Any infection	Mixture of variants	28–55	90 (80 to 95)
						56–83	89 (83 to 93)
						84–111	85 (78 to 90)
						112–139	90 (67 to 97)
						>139	90 (59 to 98)
			≥18 years	Severe disease	Delta	28–55	98 (93 to 100)
						56–83	98 (95 to 99)
						84–111	99 (94 to 100)
						112–139	92 (66 to 98)
			≥70 years	Severe disease	Mixture of variants	28–55	97 (81 to 100)
						56–83	..
						84–111	..
						112–139	93 (45 to 99)
		Pfizer–BioNTech (Comirnaty)	≥18 years	Any infection	Delta	28–55	90 (89 to 91)
						56–83	88 (87 to 89)
						84–111	85 (84 to 86)
						112–139	89 (87 to 90)
						140–167	92 (89 to 94)
						168–195	76 (57 to 87)
						>195	76 (48 to 88)
			≥70 years	Any infection	Mixture of variants	28–55	84 (78 to 88)
						56–83	88 (85 to 90)
						84–111	82 (77 to 85)
						112–139	80 (69 to 87)
						>139	68 (40 to 83)
			≥18 years	Severe disease	Delta	28–55	99 (97 to 99)
						56–83	98 (97 to 99)
						84–111	95 (93 to 97)
						112–139	97 (92 to 99)
						140–167	98 (87 to 100)
			≥70 years	Severe disease	Mixture of variants	28–55	94 (88 to 97)
						56–83	95 (93 to 97)
						84–111	94 (91 to 96)
						112–139	94 (84 to 98)
Skowronski et al (Canada)^31^	Test negative case-control study (age, sex, week of analysis period, and region)	Moderna (mRNA-1273)	≥18 years	Any infection	Delta	28–55	94 (93 to 95)
						56–83	91 (90 to 93)
						84–111	88 (86 to 90)
						112–139	83 (76 to 88)
						140–167	89 (76 to 95)
						>167	80 (73 to 85)
			≥70 years	Any infection	Mixture of variants	28–55	96 (91 to 98)
						56–83	94 (92 to 96)
						84–111	93 (90 to 95)
						112–139	85 (75 to 91)
						>139	72 (51 to 84)
			≥18 years	Severe disease	Delta	28–55	99 (96 to 100)
						56–83	98 (95 to 99)
						84–111	96 (92 to 98)
						112–139	84 (63 to 93)
			≥70 years	Severe disease	Mixture of variants	28–55	..
						56–83	98 (95 to 99)
						84–111	96 (92 to 98)
						112–139	81 (56 to 92)
		Pfizer–BioNTech (Comirnaty)	≥18 years	Any infection	Delta	28–55	92 (92 to 93)
						56–83	90 (90 to 91)
						84–111	89 (88 to 90)
						112–139	86 (81 to 89)
						140–167	77 (67 to 84)
						168–195	83 (79 to 86)
						>195	80 (76 to 84
			≥70 years	Any infection	Mixture of variants	28–55	91 (88 to 93)
						56–83	91 (89 to 92)
						84–111	91 (89 to 92)
						112–139	91 (86 to 94)
						>139	72 (54 to 83)
			≥18 years	Severe disease	Delta	28–55	99 (98 to 99)
						56–83	98 (97 to 98)
						84–111	97 (96 to 98)
						112–139	98 (88 to 100)
						140–167	92 (41 to 99)
						>167	98 (91 to 99)
			≥70 years	Severe disease	Mixture of variants	28–55	96 (93 to 98)
						56–83	97 (96 to 98)
						84–111	96 (94 to 97)
						112–139	96 (89 to 99)
						>139	98 (83 to 100)
Tenforde et al (USA)^32^	Test negative case-control study (age, sex, admission date, and race or ethnicity)	Moderna (mRNA-1273)	≥18 years	Severe disease	Mixture of variants	14–120	91 (87 to 93)
						>120	85 (77 to 91)
		Pfizer–BioNTech (Comirnaty)	≥18 years	Severe disease	Mixture of variants	14–120	85 (82 to 88)
						>120	64 (51 to 73)
Irizarry et al (Puerto Rico and USA)^33^	Retrospective cohort (age, sex, and time-varying incidence rates)	Janssen (Ad26.COV2.S)	≥18 years	Any infection	Mixture of variants	Day 14	62 (54 to 68)‖
						Day 172	36 (30 to 42)‖
			≥18 years	Severe disease	Mixture of variants	Day 14	81 (60 to 91)‖
						Day 172	67 (53 to 76)‖
		Moderna (mRNA-1273)	≥18 years	Any infection	Mixture of variants	Day 14	90 (88 to 91)‖
						Day 144	73 (70 to 76)‖
			≥18 years	Severe disease	Mixture of variants	Day 14	95 (89 to 97)‖
						Day 144	90 (84 to 94)‖
		Pfizer–BioNTech (Comirnaty)	≥12 years	Any infection	Mixture of variants	Day 14	87 (85 to 89)‖
						Day 151	57 (53 to 60)‖
			≥12 years	Severe disease	Mixture of variants	Day 14	92 (85 to 95)‖
						Day 151	80 (73 to 85)‖
Poukka et al (Finland)^34^	Retrospective cohort (age, sex, presence of medical conditions predisposing to severe COVID-19, and residence in the most affected district)	Vaxzevria (AstraZeneca)	18–69 years	Any infection	Delta‡	14–90	88 (71 to 95)
						91–180	62 (177 to 95)
			18–69 years	Severe disease	Delta‡	14–90	100 (25 to 100)¶
						91–180	81 (9 to 96)
		Moderna (mRNA-1273)	17–69 years	Any infection	Mixture of variants	14–90	84 (68 to 92)
						91–180	69 (124 to 96)
		Pfizer–BioNTech (Comirnaty)	17–69 years	Any infection	Mixture of variants	14–90	83 (80 to 85)
						91–180	63 (56 to 69)
						>180	55 (45 to 64)
			17–69 years	Severe disease	Mixture of variants	14–90	99 (97 to 100)
						91–180	98 (91 to 99))
						>180	98 (89 to 100)
Hall et al (UK)^35^	Prospective cohort (age, gender, ethnicity, comorbidities, workplace setting, contact with COVID-19 patients, region, time since vaccination, and previous infection status)	Vaxzevria (AstraZeneca)	≥18 years	Any infection	Mixture of variants	14–73	49 (16 to 69)
						74–133	47 (26 to 63)
						>133	51 (18 to 71)
		Pfizer–BioNTech (Comirnaty)	≥18 years	Any infection	Mixture of variants	14–73	81 (68 to 89)
						74–133	65 (56 to 73)
						134–193	67 (58 to 75)
						>193	43 (17 to 61)

* For each study, definitions for symptomatic and severe disease are included in the appendix (pp 19–20).

† On the basis of sequencing or genotyping unless otherwise noted.

‡ Vaccine efficacy or effectiveness was assessed during the period of variant predominance as determined by background surveillance; sequencing to determine specific variants was not performed on individual study cases.

§ Time intervals correspond to the following periods in publication: May and April, late March, early March, late February, early February, and late January (for any infection) and May and April, late March, early March and late February, early February and late January (for severe disease). Intervals represent the full range of possible durations an individual could have been fully vaccinated on the basis of the period of vaccination and dates of testing.

¶ 95% CIs were not provided in the publication because there were no COVID-19 cases in the vaccinated group. The lower limit of the 95% CI was calculated for the purpose of this review to allow for inclusion in the meta-regression. The methods are described in the appendix (pp 13–14).

‖ Estimates include 99% CIs as reported in the publication.

**Figure 2:**
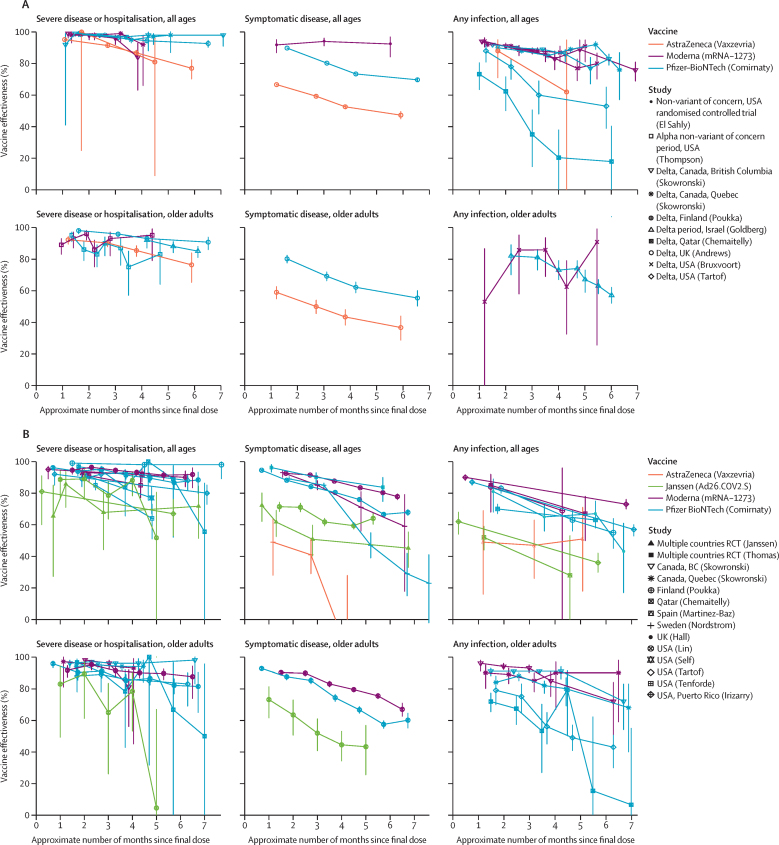
Duration of vaccine effectiveness for single-variant or non-variant-of-concern settings or mixed-variant settings Duration of vaccine effectiveness for single-variant or non-variant-of-concern settings (A) or mixed-variant settings (B). The lower bound of 95% CIs when vaccine efficacy or effectiveness is equal to 100% were undefined in manuscripts (n=1 in panel A and n=2 in panel B), and are shown here approximated (appendix pp 13–14).

Ten studies evaluated the vaccine efficacy or effectiveness over time for SARS-CoV-2 infection, among which were 26 vaccine-specific analyses (Pfizer–BioNTech-Comirnaty, n=13; Moderna-mRNA-1273, n=9; Janssen-Ad26.COV2.S, n=2; AstraZeneca-Vaxzevria, n=2; table 1).^10^, ^22^, ^23^, ^26^, ^28^, ^31^, ^33^, ^34^, ^35^ Ten vaccine-specific analyses took place in single-variant settings (all delta settings), and 16 in mixed-variant settings. 18 vaccine-specific analyses included people of all ages and eight analyses were done among older people. Among the 26 vaccine-specific analyses of vaccine efficacy or effectiveness for SARS-CoV-2 infection, the majority (22 [85%] of 26) showed at least a 10·0 percentage point decrease from peak vaccine efficacy or effectiveness and ten (38%) analyses showed at least a 25·0 percentage point drop from peak efficacy or effectiveness (table 2). Declines in vaccine efficacy or effectiveness against infection were observed in both variant settings, in both age groups, and for all four vaccines (figure 2A, figure 2B). When combining all vaccine efficacy or effectiveness evaluations of SARS-CoV-2 infection, regardless of variant type, in the meta-regression the vaccine efficacy or effectiveness decreased on average by 21·0 percentage points (95% CI 13·9–29·8) among people of all ages and by 20·7 percentage points (10·2–36·6) among older people, between 1 month and 6 months after the final vaccine dose.

**Table 2:** Assessment and meta-regression on the duration of vaccine efficacy and effectiveness

		Number of vaccine-specific analyses (number of studies)*	**Vaccines evaluated**	**Percentage point decrease from peak vaccine efficacy or effectiveness**		**Decrease in percentage points in vaccine efficacy or effectiveness from 1 month to 6 months after final dose (95% CI)**†			
				≥10%	≥25%	Stratified by variant context	p value	Combined variant contexts	p value
**SARS-CoV-2 infection**									
All ages									
	Single or non-VOC	Eight analyses (six studies)	Pfizer–BioNTech (Comirnaty; n=4), Moderna (mRNA-1273; n=3), and AstraZeneca (Vaxzevria; n=1)	7 (88%)	3 (38%)	18·0 (8·0 to 33·9)	p=0·0008	21·0 (13·9 to 29·8)	p<0·0001
	Mixture of variants	Ten analyses (four studies)	Pfizer–BioNTech (Comirnaty; n=4), Moderna (mRNA-1273; n=3), Janssen (Ad26.COV2.S; n=2), and AstraZeneca (Vaxzevria; n=1)	8 (80%)	4 (40%)	23·3 (12·1 to 38·1)	p=0·0003	..	..
Older adults‡									
	Single or non-VOC	Two analyses (two studies)	Pfizer–BioNTech (Comirnaty; n=1) and Moderna (mRNA-1273; n=1)	2 (100%)	1 (50%)	30·9 (−8·8 to 100)§	p=0·13	20·7 (10·2 to 36·6)	p=0·0004
	Mixture of variants	Six analyses (four studies)	Pfizer–BioNTech (Comirnaty; n=4) and Moderna (mRNA-1273; n=2)	5 (83%)	2 (33%)	18·1 (7·5 to 35·1)	p=0·003	..	..
**COVID-19 symptomatic disease**									
All ages									
	Single or non-VOC	Three analyses (two studies)	Pfizer–BioNTech (Comirnaty; n=1), Moderna (mRNA-1273; n=1), and AstraZeneca (Vaxzevria; n=1)	2 (66%)	0	22·2 (−7·0 to 100)§	p=0·12	24·9 (13·4 to 41·6)	p<0·0001
	Mixture of variants	Eight analyses (four studies)	Pfizer–BioNTech (Comirnaty; n=3), Moderna (mRNA-1273; n=2), AstraZeneca (Vaxzevria; n=1), and Janssen (Ad26.COV2.S; n=2)	8 (100%)	5 (63%)	27·8 (13·0 to 51·5)	p=0·0005	..	..
Older adults‡									
	Single or non-VOC	Two analyses (one study)	Pfizer–BioNTech (Comirnaty; n=1) and AstraZeneca (Vaxzevria; n=1)	2 (100%)	0	27·1 (−20·1 to 100)§	p=0·14	32·0 (11·0 to 69·0)	p=0·006
	Mixture of variants	Three analyses (one study)	Pfizer–BioNTech (Comirnaty; n=1), Moderna (mRNA-1273; n=1), and Janssen (Ad26.COV2.S; n=1)	3 (100%)	3 (100%)	36·1 (16·3 to 70·5)	p=0·008	..	..
**COVID-19 severe disease**									
All ages									
	Single or non-VOC	Eight analyses (five studies)	Pfizer–BioNTech (Comirnaty; n=4), Moderna (mRNA-1273; n=2), and AstraZeneca (Vaxzevria; n=2)	3 (38%)	0	7·8 (5·3 to 11·1)	p<0·0001	10·0 (6·1 to 15·4)	p<0·0001
	Mixture of variants	14 analyses (seven studies)	Pfizer–BioNTech (Comirnaty; n=7), Moderna (mRNA-1273; n=4), and Janssen (Ad26.COV2.S; n=3)	8 (57%)	2 (14%)	9·9 (4·8 to 17·1)	p=0·0001	..	..
Older adults‡									
	Single or non-VOC	Five analyses (three studies)	Pfizer–BioNTech (Comirnaty; n=3), Moderna (mRNA-1273; n=1), and AstraZeneca (Vaxzevria; n=1)	2 (40%)	0	11·8 (3·4 to 28·1)	p=0·008	9·5 (5·7 to 14·6)	p<0·0001
	Mixture of variants	Nine analyses (five studies)	Pfizer–BioNTech (Comirnaty; n=5), Moderna (mRNA-1273; n=3), and Janssen (Ad26.COV2.S; n=1)	4 (44%)	2 (22%)	7·7 (2·7 to 15·8)	p=0·0032	..	..

VOC=variant of concern.

* One Canadian study is counted twice because it reported results for two provinces separately.

† Obtained from the meta-regression modeling log (1 – vaccine efficacy or effectiveness) regressed on log (months after final dose).

‡ Older people, as defined in the study and at least 50 years of age.

§ Set to 100% when the upper limit exceeded 100%.

Six studies evaluated the vaccine efficacy or effective-ness over time for symptomatic COVID-19 disease, among which there were 16 vaccine-specific analyses (Pfizer–BioNTech-Comirnaty, n=6; Moderna-mRNA-1273, n=4; Janssen-Ad26.COV2.S, n=3; and AstraZeneca-Vaxzevria, n=3; table 1).^19^, ^20^, ^21^, ^25^, ^29^, ^30^ Five vaccine-specific analyses took place in single-variant settings (four in delta settings and one in non-VOC settings), and 11 took place in mixed-variant settings. 11 vaccine-specific analyses were done among people of all ages and five among older people. Among the 16 vaccine-specific analyses of vaccine efficacy or effectiveness for symptomatic disease, the majority (15 [94%] of 16) showed at least a 10·0 percentage point decrease from the peak vaccine efficacy or effectiveness and eight (50%) showed at least a 25·0 percentage point drop, all of which were in mixed-variant settings (table 2). Declines in vaccine efficacy or effectiveness against symptomatic disease were observed in both variant settings, in both age groups, and among all four vaccines (figure 2A, figure 2B). Of note, the one study that showed no decline in vaccine efficacy or effectiveness was the extended follow-up of the randomised controlled trial of the Moderna-mRNA-1273 vaccine during a period of non-VOC circulation in the USA.^19^ When combining all vaccine efficacy or effectiveness evaluations of symptomatic disease, regardless of variant type, in the meta-regression the vaccine efficacy or effectiveness decreased on average by 24·9 percentage points (95% CI 13·4–41·6) in people of all ages and by 32·0 percentage points (11·0–69·0) in older people, between 1 month and 6 months after the final vaccine dose.

12 studies evaluated the vaccine efficacy or effectiveness over time for severe COVID-19 disease, among which there were 36 vaccine-specific analyses (Pfizer–BioNTech-Comirnaty, n=19; Moderna-mRNA-1273, n=10; Janssen-Ad26.COV2.S, n=4; AstraZeneca-Vaxzevria, n=3; table 1).^10^, ^21^, ^22^, ^24^, ^25^, ^27^, ^28^, ^29^, ^31^, ^32^, ^33^, ^34^ 13 vaccine-specific analyses took place in single-variant settings (11 in delta settings and two in Alpha settings), and 23 analyses took place in mixed-variant settings. 22 vaccine-specific analyses were done among people of all ages and 14 among older people. Among the 36 vaccine-specific analyses of vaccine efficacy or effectiveness for severe disease, 17 (47%) showed at least a 10·0 percentage point decrease from the peak vaccine efficacy or effectiveness (table 2). Four (11%) vaccine-specific analyses showed at least a 25·0 percentage point decrease in vaccine efficacy or effectiveness; two analyses from one study in Qatar for Pfizer–BioNTech-Comirnaty and the other two analyses from a study in the USA for Janssen-Ad26.COV2.S.^22^, ^29^ In both studies, the decrease of at least 25·0 percentage points in vaccine efficacy or effectiveness was observed among both age categories in mixed-variant settings, with wide 95% CIs for the lowest vaccine efficacy or effectiveness estimates. Seven (19%) vaccine-specific analyses (from five studies) showed a decrease in estimates of absolute vaccine efficacy or effectiveness against severe disease to less than 70% at a single timepoint in follow-up (Pfizer–BioNTech-Comirnaty, n=3; and Ad26.COV2.S, n=4; figure 2A, figure 2B).^21^, ^22^, ^27^, ^32^, ^33^ When combining all vaccine efficacy or effectiveness evaluations of severe disease, regardless of variant type, in the meta-regression the vaccine efficacy or effectiveness decreased on average by 10·0 percentage points (95% CI 6·1–15·4) among people of all ages and by 9·5 percentage points (5·7–14·6) among older people between 1 month and 6 months after the final vaccine dose.

**Figure 3:**
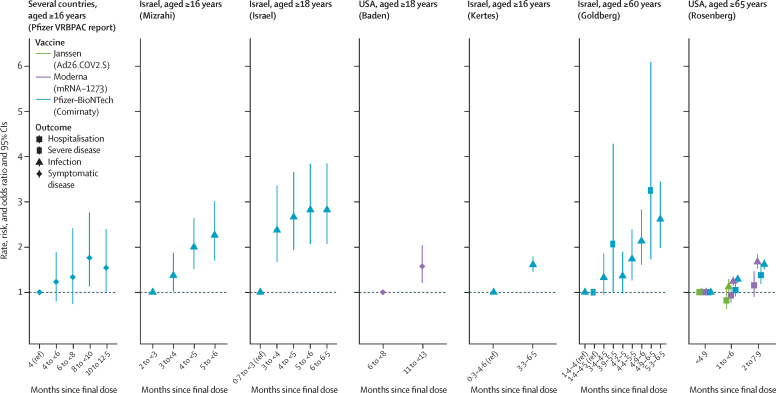
Rate, risk, and odds ratios of COVID-19 breakthrough cases caused by the delta variant by time of vaccination X axis values overlap because of data availability in cited references.

In the analysis of delta breakthrough infections, we found seven studies through the search strategy, and one study through searching regulatory applications; one study was excluded because it combined the results of several vaccines, leaving seven studies for final inclusion (figure 1 and table 3). One study had low overall risk of bias, two studies had moderate risk, and four studies had serious risk (appendix p 16). In two clinical trials, people who were initially randomly assigned to study vaccine had an increased rate of breakthrough symptomatic COVID-19 disease during the period of July, 2021, to August, 2021, when the delta variant was pre-dominant, compared with those who initially received placebo and later crossed over to receive the actual COVID-19 vaccine. This increased rate was 1·76 times (95% CI 1·13–2·76) higher for Pfizer–BioNTech-Comirnaty and 1·57 times (1·21–2·04) higher for Moderna-mRNA-1273 (figure 3).^36^, ^37^ Four observational studies in Israel of Pfizer–BioNTech-Comirnaty measured incidence after June, 2021, when delta was the predominant variant.^10^, ^16^, ^17^, ^18^ All four studies found risk of breakthrough infections that were higher among at least one cohort of people who were vaccinated further back in time than more recently vaccinated people, with increased risk of breakthrough infections ranging from 1·37 times (95% CI 1·02–1·82)^16^ to 2·82 times (2·07–3·85) higher.^17^ A study from the USA found a higher risk of breakthrough infections among people aged 65 years or older vaccinated further back in time for Pfizer–BioNTech-Comirnaty (incidence risk ratio 1·62, 95% CI 1·51–1·73) and Moderna-mRNA-1273 vaccines (incidence risk ratio 1·67, 1·52–1·84).^38^ Two studies evaluated breakthrough severe infections; one study in Israel had a maximum of 3·25 times (95% CI 1·73–6·09) increased risk of breakthrough severe infections among people aged 60 years or older, vaccinated with Pfizer–BioNTech-Comirnaty further back in time, and one study in the USA had a maximum of 1·38 times (1·18–1·62) increased risk of break-through infections among people aged 65 years and older who were hospitalised and vaccinated with Pfizer–BioNTech-Comirnaty further back in time.^10^, ^38^

**Table 3:** Characteristics of SARS-CoV-2 breakthrough infection studies during periods of delta predominance

	**Study design**	**Study population (number analysed)**	**Testing period**	**Vaccine**	**Age group**	**Disease outcome**	**Days since final dose**	**Number of cases**	**Total number of persons or person-years**	Risk or rate*per 1000	**Risk ratio, rate ratio, or odds ratio (95% CI)**
Goldberg et al (Israel)^10^	Retrospective cohort	936 711 vaccinated Israeli residents aged ≥60 years	July 11, 2021, to July 31, 2021	Pfizer–BioNTech (Comirnaty)	≥60 years	Any infection	41–121†	51	40 111	1·27	1·00 (ref)
							102–137†	105	62 317	1·68	1·33 (0·95–1·85)
							128–152†	107	61 886	1·73	1·36 (0·97–1·90)
							133–166†	148	67 028	2·21	1·74 (1·26–2·39
							147–180†	973	358 592	2·71	2·13 (1·61–2·83)
							161–196†	2348	706 990	3·32	2·61 (1·98–3·45)
						Severe disease	41–137‡	10	102 428	0·10	1·00 (ref)
							118–166‡	26	128 914	0·20	2·07 (1·00–4·28)
							147–196‡	338	1 065 582	0·32	3·25 (1·73–6·09)
Kertes et al (Israel)^16^	Retrospective cohort	1 423 098 vaccinated members of the Maccabi health-care system, aged ≥16 years	June 9, 2021, to July 18, 2021	Pfizer–BioNTech (Comirnaty)	≥16 years	Any infection	9–139	..	601 867	..	1·00 (ref)
							101–198	..	821 231	..	1·61 (1·45–1·79)§
Israel et al (Israel)^17^	Retrospective cohort	33 993 vaccinated members of Leumit Health Services, aged ≥18 years	May 15, 2021, to July 26, 2021	Pfizer–BioNTech (Comirnaty)	≥18 years	Any infection	21–89	6320 (across all intervals)	37 920 (across all intervals)	..	1·00 (ref)
							90–119			..	2·37 (1·67–3·36)§
							120–149			..	2·66 (1·94–3·66)§
							150–179			..	2·82 (2·07–3·84)§
							≥180			..	2·82 (2·07–3·85)§
Mizrahi et al (Israel)^18^	Retrospective cohort	1 352 444 vaccinated members of Maccabi Healthcare Services, aged ≥16 years	June 1, 2021, to July 27, 2021	Pfizer–BioNTech (Comirnaty)	≥16 years	Any infection	60–89¶	76	44 734	..	1·00 (ref)
							90–119¶	858	371 929	..	1·37 (1·02–1·82)§
							120–149¶	1550	460 500	..	2·00 (1·51–2·64)§
							150–179¶	1736	475 281	..	2·26 (1·70–3·01)§
Baden et al (USA)^36^	Randomised controlled trial crossover	26 177 vaccinated participants, aged ≥18 years	July 1, 2021, to Aug 27, 2021	Moderna (mRNA-1273)	≥18 years	Any symptomatic disease	180–239‖	88	1796 person-years	49·0	1·00 (ref)
							330–389‖	162	2102 person-years	77·1	1·57 (1·21–2·04)
Pfizer VRBPAC report (several countries)^37^	Randomised controlled trial crossover	36 442 vaccinated participants, aged ≥18 years	July 1, 2021, to Aug 31, 2021	Pfizer–BioNTech (Comirnaty)	≥18 years	Any symptomatic disease	<120	26	598 person-years	43·510	1·00 (ref)
							121–179	108	2019 person-years	53·502	1·23 (0·80–1·89)
							180–239	19	327 person-years	58·117	1·34 (0·74–2·41)
							240–299	73	951 person-years	76·733	1·76 (1·13–2·76)
							≥300	86	1282 person-years	67·082	1·54 (1·00–2·39)
Rosenberg et al (USA)^38^	Prospective cohort	6 394 228 vaccinated residents of New York State, aged ≥18 years and 2 123 651 residents aged ≥65 years	May 1, 2021, to Sept 3, 2021	Janssen (Ad26.COV2.S)	≥65 years	Any infection	1–149**	405	65 182	6·21	1·00 (ref)
							31–180**	342	49 109	6·96	1·12 (0·97–1·29)
					≥65 years	Hospitalisation	1–149**	141	65 182	2·16	1·00 (ref)
							31–180**	87	49 109	1·77	0·82 (0·63–1·07)
				Moderna (mRNA-1273)	≥65 years	Any infection	1–149**	1189	437 431	2·72	1·00 (ref)
							31–180**	1439	426 802	3·37	1·24 (1·15–1·34)
							62–239**	644	141 769	4·45	1·67 (1·52–1·84)
					≥65 years	Hospitalisation	1–149**	238	437 431	0·54	1·00 (ref)
							31–180**	216	426 802	0·51	0·92 (0·77–1·12)
							62–239**	89	141 769	0·63	1·15 (0·90–1·47)
				Pfizer–BioNTech (Comirnaty)	≥65 years	Any infection	1–149**	1898	427 979	4·43	1·00 (ref)
							31–180**	1961	343 396	5·71	1·29 (1·21–1·37)
							62–239**	1411	196 823	7·17	1·62 (1·51–1·73)
					≥65 years	Hospitalisation	1–149**	391	427 979	0·91	1·00 (ref)
							31–180**	329	343 396	0·95	1·05 (0·91–1·21)
							62–239**	248	196 823	1·26	1·38 (1·18–1·62)

* Person-time and person counts as denominators allowed us to calculate rate ratios and risk ratios, respectively. Rates from crossover studies of randomised controlled trials are those presented in publications; all other risks are crude ratios calculated from the raw data for the purpose of this analysis, with the exception of Kertes and colleagues,^16^ Israel and colleagues,^17^ and Mizrahi colleagues,^18^ for which adjusted odds ratios were available and are included in the table.

† Time intervals correspond with groups vaccinated in May, April, and late March, in early March and late February, and in early February and late January, as noted in the publication. May and April groups were combined for the purpose of this analysis.

‡ Time intervals correspond to the groups vaccinated in May, April, and late March, early March and late February, and early February and late January. Groups were combined for the purpose of this analysis.

§ Adjusted odds ratios from publications. For Kertes and colleagues, odds ratios were adjusted for age group, socioeconomic status, and presence of chronic illnesses (heart disease, hypertension, diabetes, chronic kidney disease, and immunosuppressive disorder). For Israel and colleagues, odds ratios were adjusted for age, sex, socioeconomic status, and comorbid conditions. For Mizrahi and colleagues, odds ratios were adjusted for comorbidities after matching for age group, sex, city of residence, and socioeconomic status.

¶ 60–89 days, 90–119 days, 120–149 days, and 150–179 days correspond to April, March, February, and January vaccine cohorts in the study.

‖ 180–239 days corresponds to the study period of Dec 29, 2020, to Apr 30, 2021 (open-label phase); 330–389 days corresponds to the study period of July 27, to Dec 16, 2020 (masked phase).

** 1–149 days, 31–180 days, and 62–239 days correspond to people vaccinated in April, March, and January and February, respectively, as noted in the publication.

Among the 18 included studies, three had low overall risk of bias, eight had moderate risk, and seven had serious risk (appendix p 16). The major domain of bias was incomplete adjustment for confounders. Several particular biases can influence the results when assessing the duration of vaccine efficacy or effectiveness over time (table 4).

**Table 4:** Biases that can affect estimates of duration of vaccine effectiveness for COVID-19 vaccines

	**Examples**	**How to minimise bias**
People who are unvaccinated have a differential risk of exposure as coverage plateaus at a high level	Demographic and ethnic high-risk groups are over-represented in unvaccinated groups	Adjust for factors if measured and consider using a vaccinated group as a comparator
Earliest vaccinated groups have sustained higher risk	Health-care workers and care home residents	Adjust for factors if measured and stratify vaccine effectiveness analysis by phase of vaccine introduction
People who are vaccinated change behaviour over time in a way that is different to those who are unvaccinated	Differential adherence to NPIs and restrictions by vaccine status (eg, Green Pass or vaccine passports)	Adjust for NPI adherence alone or with mobility (not possible if using administrative databases)
People who are vaccinated have differential testing behaviour over time relative to those who are unvaccinated	Testing differs by vaccine status (eg, Green Pass or vaccine passports), travel-related testing, and use of home testing (eg, lateral flow tests) before accessing confirmatory tests	Test-negative design adjust for testing frequency in the analysis and exclude PCR-negative tests if they shortly follow lateral flow positive tests
Vaccine-derived immunity increases among people who are unvaccinated	Depletion of susceptible people because of higher rates of infection in those who are unvaccinated over time; this depletion is only an issue if the additional protection of vaccine in people with past infection is greater than those not previously infected	Test (or ask about) previous infection and exclude people with infection from analysis
Misclassification of COVID-19 deaths increases with time	Older people are more likely to die of all causes with time	Verify cause of death where possible
Denominator overestimation of people who are unvaccinated over time	Emigration of people initially in the cohort study out of the catchment area	Regularly correct denominator in cohort studies
Changes in positive predictive value of a COVID-19-positive test result	When prevalence is low for the same specificity, positivity predictive value will be lower, leading to a greater misclassification bias	Use tests with high positive predictive values and use symptomatic cases
Changes in interval between doses over time	Some countries changed dosing intervals several times because of vaccine supply fluctuations	Assess whether interval affects vaccine effectiveness in sensitivity analyses and consider restricting the analysis to the dominant dosing interval

NPIs include mask wearing. NPI=non-pharmaceutical interventions.

## Discussion

We showed that the decline in vaccine efficacy or effectiveness against severe COVID-19 disease with time since vaccination was less than that for SARS-CoV-2 infection and symptomatic COVID-19 disease. In most studies, the vaccine efficacy or effectiveness against severe disease remained high (≥70%) for up to 6 months after vaccination for all four vaccines that we evaluated (and mostly ≥80% for the two mRNA vaccines). Nonetheless, by 6 months there was a drop in vaccine efficacy or effectiveness for severe disease of a mean of 9·5–10·0 percentage points, including among older people. This smaller decrease in vaccine efficacy or effectiveness for severe disease is reassuring given that prevention of severe disease and death remains the primary objective of COVID-19 vaccination. By contrast, most studies showed a notable decrease in vaccine efficacy or effectiveness by 6 months after vaccination for SARS-CoV-2 infection (a decrease of 21 percentage points) and all symptomatic COVID-19 disease (a decrease of 25–32 percentage points). However, the data were heterogenous, with some studies showing minimal decrease in vaccine efficacy or effectiveness over time and others showing substantial decrease (ie, ≥25 percentage points).

A decrease in the vaccine efficacy or effectiveness over time has three potential explanations: the decrease can reflect lower vaccine efficacy or effectiveness against a new variant; true waning immunity caused by loss of vaccine-induced immunological protection; or bias. We showed that vaccine efficacy or effectiveness decreased over time when restricting analysis to a single variant. This finding was reinforced by our second analysis of breakthrough infections with the delta variant that showed higher breakthrough risk with longer times since vaccination. Together these findings suggest that the decrease in vaccine efficacy or effectiveness over time was likely not caused, for the most part, by the temporal increase in prevalence of the delta variant.

Waning vaccine efficacy or effectiveness is a plausible explanation for the decrease in vaccine efficacy or effectiveness against infection and disease. The finding is consistent with immunological data showing that over time, amounts of most vaccine-derived antibodies, including those that neutralise the virus, decline.^39^, ^40^ Yet, because the immune system forms memory cells that can be activated upon exposure to a virus and includes cellular immunity, it is not clear whether this observed antibody decay results in diminished vaccine efficacy or effectiveness, and if so, over what timeframe and against which outcomes. Nevertheless, further support for possible waning immunity comes from evidence showing that after giving a booster dose the vaccine efficacy or effectiveness increases compared with people who had only received the primary vaccine series.^41^, ^42^ Moreover, it has been shown that with increasing time since full vaccination, the viral load of breakthrough infections increases, but becomes lower again soon after booster vaccination.^43^ We did not see an obvious difference in the magnitude or timing of decrease in vaccine efficacy or effectiveness between people of all ages and older people in the meta-regression, although the number of studies was probably too low to make definitive conclusions. A study from the UK showed that decreases in vaccine efficacy or effectiveness seemed to occur more among clinically extremely vulnerable older people.^25^

Although waning immunity is consistent with the data, we cannot exclude the possibility that the observed decrease in vaccine efficacy or effectiveness over time was caused, either partly or wholly, by biases. An underlying assumption of observational studies is that people who are unvaccinated should be at the same risk of exposure to SARS-CoV-2 as those who are vaccinated in the same population. At high vaccine coverage, this assumption might no longer apply, given that people who remain unvaccinated either choose to remain unvaccinated or are unable to get vaccinated for reasons that might be associated with a differential risk of COVID-19 compared with the general population.^30^, ^44^, ^45^, ^46^ Although some differences can be identified and adjusted for in the analysis (eg, age and demographic group), others might be less obvious, harder to measure and adjust for, and could lead to underestimation of true vaccine efficacy or effectiveness over time (eg, clinically extremely vulnerable status).^25^ The expected bias based on the magnitude and direction of the differential risk of COVID-19 among people who are unvaccinated showed that confounding is more important when the true vaccine efficacy or effectiveness is not as high (appendix p 17); this finding implies that confounding by risk among the unvaccinated group is accentuated when the vaccine has lower initial efficacy and when the true vaccine effectiveness has become lower over time.

Several other potential biases in assessing the duration of vaccine efficacy or effectiveness over time can occur. Some important biases that could result in an overestimation of decreases in vaccine efficacy or effectiveness over time are as follows: the people who are vaccinated the earliest are at sustained increased risk of infection compared with those who were vaccinated later; people who are vaccinated change their behaviour and testing frequency over time increasing the likelihood of being infected or being detected as infected, particularly with increased mobility for those who can show vaccination status; and people who remain unvaccinated have increased infection-derived immunity leading to spurious interpretations of reductions in vaccine efficacy or effectiveness as waning protection.^47^ Because most of these biases are unmeasured, we cannot definitely establish which ones most affected the studies included in this analysis.

Our systematic review had several other potential limitations. First, given the rapid pace and multiple preprint publishing options for COVID-19-related content, it is possible that additional studies on vaccine duration of protection were not captured by our search strategy, and new studies will become available after our cutoff date. Second, many preprint studies included in this analysis could have their data changed in the eventual publication. Third, insufficient studies met our inclusion criteria to allow for meaningful comparisons between different vaccine platforms. Fourth, a small number of vaccines were evaluated, and from few geographical settings, which might not be represen-tative of other settings with different epidemiological conditions in which duration of vaccine protection might differ (eg, more or less previous infection). Fifth, few studies evaluated vaccine efficacy or effectiveness separately in younger people; the three studies that did so showed similar patterns of decrease in vaccine efficacy or effectiveness over time to that seen in adults of all ages and older people (appendix p 18). Sixth, no heterologous schedules were evaluated. Seventh, all included studies were published before the emergence and spread of the omicron variant. Lastly, we based our calculations on published or derived estimates of vaccine efficacy or effectiveness and their SEs rather than original person-level event data. One manifestation of this limitation is the necessity to introduce small adjustments to vaccine efficacy or effectiveness estimates of 100% to include these estimates in our model for the log-transformed relative-risk estimates. The potential bias in the summary vaccine efficacy or effectiveness estimates is small because there were only three vaccine effectiveness estimates of 100%, and two had wide CIs, which decreases their contribution in the regression model.

Further follow-up of vaccine efficacy or effectiveness against severe disease, the outcome that drives most COVID-19 policy decisions, for all vaccines beyond 6 months is needed to clarify how much more waning of protection might occur with longer duration since full vaccination.^48^ Continuing to produce reliable and vaccine-specific vaccine efficacy or effectiveness estimates over extended periods of time after vaccination against multiple outcomes, and in the setting of emerging variants against which vaccine efficacy or effectiveness might be lower and waning occurs faster, such as the omicron variant, is crucial for COVID-19 vaccine policy and decision-making bodies.^49^ Policy makers considering the use and timing of booster doses should integrate vaccine-specific and outcome-specific evidence of decreasing vaccine efficacy or effectiveness with other considerations, such as vaccine coverage and supply, prioritisation relative to primary-series vaccination, programmatic issues, and local COVID-19 epidemiology.

## Data sharing

All data included were derived from publicly available documents cited in the references. Extracted data are available upon request to the corresponding author.

## Declaration of interests

MMH reports research grants from WHO, the Coalition for Epidemic Preparedness Innovations (CEPI), the Asian Development Bank (ADB), the Bill & Melinda Gates Foundation, and Pfizer (all paid to the institution). RA reports a contract from the US Centers for Disease Control and Prevention, a grant from the Chile Ministry of Science, and consulting fees from the Mayo Clinic and Chile Ministry of Health. YG reports research grants from the United States-Israel Binational Science Foundation (BSF) and the Israel Science Foundation. MJG reports research grants from the South African Medical Research Council and the Gates Foundation (all paid to the institution) and participation on a data safety monitoring board for a study on the effectiveness of COVID-19 vaccination against SARS-CoV-2-associated hospitalisation and death. AH reports research grants from United States-Israel Binational Science Foundation. KLO'B serves as the Secretariat for the WHO Strategic Advisory Group of Experts on Immunisation. MDK reports research grants from WHO, CEPI, ADB, and Pfizer (all paid to the institution) and consultancy fees from Merck.
