
JOURNAL INFORMATION
==============================
NLM Title Abbreviation: Lancet
Journal ID: Lancet
NLM Journal ID: 2985213R

Lancet (London, England)

ISSN: 0140-6736
EISSN: 1474-547X

ARTICLE INFORMATION
==============================
PMCID: PMC8979561
PMID: 35390323
DOI: 10.1016/S0140-6736(22)00428-7
Article ID: S0140-6736(22)00428-7
Article version: 2
Subjects: Department of Error

Department of Error

Print publication date: 2024 Aug 31
Volume: 404
Issue: 10455
First page: e3
Last page: e3
Copyright: .
Copyright year: 2024
Copyright holder: Elsevier Ltd
License: This is an open access article under the CC BY license (http://creativecommons.org/licenses/by/4.0/).
License URL: https://creativecommons.org/licenses/by/4.0/

Erratum for: Duration of effectiveness of vaccines against SARS-CoV-2 infection and COVID-19 disease: results of a systematic review and meta-regression 399 10328 5 3 2022 924 944 Lancet (London, England) 10.1016/S0140-6736(22)00152-0 PMC8863502 35202601

==============================
Feikin DR, Higdon MM, Abu-Raddad LJ, et al. Duration of effectiveness of vaccines against SARS-CoV-2 infection and COVID-19 disease: results of a systematic review and meta-regression. Lancet 2022; 399: 924–44—In this Article, in table 4, in the first column, the fifth row should read “Infection-derived, immunity increases among people who are unvaccinated”. The appendix has also been corrected. These corrections have been made to the online version as of April 4, 2022.

