# Supplementary material for: Duration of effectiveness of vaccines against SARS-CoV-2 infection and COVID-19 disease: results of a systematic review and meta-regression
Source: Lancet. 2022 Mar 5;399(10328):924–44. doi: 10.1016/S0140-6736(22)00152-0 (PMC8863502; doi:10.1016/S0140-6736(22)00152-0)
Supplement: Supplementary appendix [file mmc1.pdf]

THE LANCET

Supplementary appendix

This appendix formed part of the original submission and has been peer reviewed. We post it as supplied by the authors.

Supplement to: Feikin DR, Higdon MM, Abu-Raddad LJ, et al. Duration of effectiveness of vaccines against SARS-CoV-2 infection and COVID-19 disease: results of a systematic review and meta-regression. *Lancet* 2022; published online Feb 21. https://doi.org/10.1016/S0140-6736(22)00152-0.

Duration of effectiveness of vaccines against SARS-CoV-2 infection and COVID-19 disease: Results of a systematic review and meta-regression

Daniel R. Feikin*, Melissa M. Higdon*, Laith J. Abu-Raddad, Nick Andrews, Rafael Araos, Yair Goldberg, Michelle J. Groome, Amit Huppert, Katherine L. O'Brien, Peter G. Smith, Annelies Wilder-Smith, Scott Zeger, Maria Deloria Knoll*, Minal K. Patel*

Affiliations:
Department of Immunizations, Vaccines and Biologicals, World Health Organization, Geneva, Switzerland (D. Feikin, MD, K. O'Brien, MD, A. Wilder-Smith, MD, M. Patel, MD); International Vaccine Access Center, Johns Hopkins Bloomberg School of Public Health, Baltimore, Maryland, U.S.A. (M. Higdon, MPH, M. Deloria Knoll, PhD); Infectious Disease Epidemiology Group, Weill Cornell Medicine–Qatar Doha, Qatar (L.J. Abu-Raddad, PhD); UK Health Security Agency, London, UK (N Andrews PhD); Instituto de Ciencias e Innovacion en Medicina, Facultad de Medicina, Clinica Alemana Universidad del Desarrollo, and the Advanced Center for Chronic Diseases, Santiago, Chile (R. Araos, MD); Technion - Israel Institute of Technology, Haife, Israel (Y. Goldberg, PhD); The Gertner Institute for Epidemiology & Health Policy Research, Sheba Medical Center, and Tel Aviv University, Tel Aviv, Israel (A. Huppert, PhD); National Institute for Communicable Diseases, Division of the National Health Laboratory Service, Johannesburg, South Africa and School of Pathology, Faculty of Health Sciences, University of the Witwatersrand, Johannesburg, South Africa (M. Groome, PhD); MRC International Statistics and Epidemiology Group MRC International Statistics and Epidemiology Group, London School of Hygiene and Tropical Medicine, United Kingdom of Great Britain and Northern Ireland (Prof P. G. Smith, DSc). Department of Epidemiology, Johns Hopkins, Bloomberg School of Public Health, Baltimore, Maryland, U.S.A. (Prof S. Zeger, PhD)

*contributed equally.

Summary

Background. Knowing if COVID-19 vaccine effectiveness wanes is critical to informing vaccine policy, such as the need for and timing of booster doses.

Methods. We performed a systematic review of pre-print and published article databases from June 17 to December 2, 2021. Studies with vaccine efficacy or effectiveness (VE) estimates at discrete time intervals after full vaccination and meeting pre-defined screening criteria underwent full-text review. We used random effects meta-regression to estimate the average change in VE from one-to-six months after full vaccination.

Findings. Of 13,744 studies screened, 310 underwent full text review, and 18 were included (all pre-Omicron). Risk of bias determination by ROB2 and ROBINS-I tools was low (n=3), moderate (n=8) and serious (n=7). Seventy-eight vaccine-specific VE evaluations were included (Pfizer/BioNTech-Comirnaty (n=38), Moderna-mRNA-1273 (n=23), Janssen-Ad26.COV2.S (n=9), and AstraZeneca-Vaxzevria (n=8). On average, VE against SARS-CoV-2 infection decreased from one-to-six months after full vaccination by 21·0 (95% CI 13·9-29·8) percentage points among persons of all ages and 20·7 (95% CI 10·2-36·6) percentage points among older persons (as defined by each study, at least ≥50 years old); for symptomatic COVID-19 disease, VE decreased by 24·9 (95% CI 13·4-41·6) and 32·0 (95% CI 11·0-69·0) percentage points, respectively; for severe COVID-19 disease, VE decreased by 10·0 (95% CI 6·1-15·4) and 9·5 (95% CI 5·7-14·6) percentage points, respectively. Most (81%) VE estimates against severe disease remained greater than 70% over time.

Interpretation. COVID-19 VE against severe disease remained high, although it did decrease some (9·5-10.0 percentage points) by six months after full vaccination. In contrast, VE against infection and symptomatic disease decreased approximately 20-30 percentage points by six months. The decrease in VE is likely due, at least in part, to waning immunity, although an effect of bias cannot be ruled out. Evaluating VE beyond six months will be critical for updating COVID-19 vaccine policy.

Funding. Coalition for Epidemic Preparedness Innovations

Research in Context

Evidence before this study

Approximately one year after the first introductions of COVID-19 vaccines, multiple studies have been published that assess vaccine efficacy and effectiveness (VE) after full vaccination. Several systematic reviews of COVID-19 vaccine efficacy/effectiveness studies have been published, but none focused on how VE changes with time since vaccination. We systematically reviewed the evidence for changes in COVID-19 VE with time since full vaccination for various clinical outcomes. Additionally, our review summarizes evidence for rates of breakthrough infections due to the Delta variant among vaccinated persons stratified by time since vaccination. In interpreting these studies, we discuss potential biases in evaluating changes in vaccine effectiveness with time since vaccination.

We searched for studies that evaluated VE at discrete time intervals after full vaccination from June 17 to December 2, 2021 in PubMed, Embase, medRxiv, bioRxiv, khub, Research Square, SSRN, Eurosurveillance.org, Europepmc.org and the World Health Organization COVID-19 Database (which compiles searches of over 100 databases, including Scopus, Web of Science and the grey literature). We searched for studies with multiple variations of the primary key search terms, of "COVID-19" and "SARS-CoV-2" and "vaccine" (including names of specific vaccines) and "randomized controlled trial" or "vaccine effectiveness" (including names of specific study designs). We also hand-searched regulatory agency databases. Studies were included if they presented VE estimates at discrete time intervals from full vaccination compared to unvaccinated persons for SARS-CoV-2 infection, COVID-19 symptomatic disease, or severe disease – for any

vaccine that has received Emergency Use Listing at WHO. VE estimates confined to a single variant and due to a mixture of variants were analyzed separately. Random effects meta-regression was used to estimate the average change in VE from one to six months after full vaccination. After applying exclusion criteria, we included 18 studies of VE at discrete time intervals after full vaccination and seven studies in which risk of breakthrough infection could be assessed by time of vaccination. In addition, the same search strategy was used to find studies presenting analyses of breakthrough infections, in which the rate, risk or odds of COVID-19 outcomes among different vaccine cohorts (i.e., vaccinated at different times) were included.

Added value of this study

We found during the six months after full vaccination the VE against SARS-CoV-2 infection and symptomatic COVID-19 disease decreased by approximately 20-30 percentage points, on average, for the four vaccines evaluated. In contrast, most studies showed that VE against severe disease was maintained above 70% after full vaccination, with minimal decrease through six months (approximately 9-10 percentage points). This is the first systematic review and meta-regression to date, to our knowledge, that describes the timing and magnitude of decreasing VE over time since full vaccination by disease outcome.

Implications of all the available evidence

Studies of the duration of protection of COVID-19 vaccine effectiveness as a whole indicate that vaccine effectiveness decreases more against infection and symptomatic disease than against severe disease in the six months after full vaccination. This decreasing VE is likely due, at least in part, to waning immunity. Multiple biases, however, can affect estimates of declining VE over

time. Whether VE will eventually decrease further against severe disease, and in the setting of new variants like Omicron, requires ongoing evaluation at later time points after full vaccination. Policy makers considering the need and timing of booster doses should integrate vaccine- and outcome-specific evidence of decreasing VE with other considerations, such as vaccine coverage and supply, prioritization relative to primary series vaccination, programmatic issues, and local COVID-19 epidemiology.

Introduction

Almost two years into the COVID-19 pandemic, multiple COVID-19 vaccines have received Emergency Use Listing/Authorization (EUL/EUA) by regulatory authorities and the World Health Organization (WHO) based on vaccine efficacy results from randomized controlled trials.[1] Efficacy results at the time of EUL/EUA, however, had a median follow-up time after full vaccination of only two-to-three months. Estimates of vaccine effectiveness among persons vaccinated as part of national vaccine roll-outs were similar to the efficacy results in the first few months after vaccine introduction.[2] Assessing the duration of protection for COVID-19 vaccines over longer time periods, however, requires continued monitoring. Knowing if and to what extent vaccine effectiveness wanes is critical to inform vaccine policy decisions, such as the need for, timing, and target populations for booster doses.

Several systematic reviews of COVID-19 efficacy/effectiveness studies have been published, but none evaluated the duration of protection of COVID-19 vaccines.[3–8] We systematically reviewed the evidence for the duration of protection of COVID-19 vaccines against various clinical outcomes by assessing studies that evaluate vaccine efficacy or effectiveness (henceforth referred to as VE) at various time periods after vaccination. Additionally, we evaluated rates of breakthrough infection due to Delta variant among vaccinated persons stratified by time since vaccination.

Methods

Search strategy and selection criteria

Since June 2021, the World Health Organization and International Vaccine Access Center at Johns Hopkins Bloomberg School of Public Health have been tracking the emerging evidence for COVID-19 VE and have posted their methodology and updated weekly results at the VIEW-HUB website[9]. For this systematic review, we followed PRISMA guidelines (Supplement S1). We considered peer-reviewed and pre-print studies published from June 17 to December 2, 2021. Randomized controlled trials of COVID-19 vaccine efficacy and observational studies of COVID-19 vaccine effectiveness were eligible. The following databases and pre-print servers were searched without restrictions by language of publication: PubMed, Embase, medRxiv, BioRxiv, khub, Research Square, SSRN, Eurosurveillance.org, Europepmc.org and the World Health Organization COVID-19 Database, which compiles searches of over 100 databases, including Scopus, Web of Science and the grey literature. The search strategy is described in Supplement S2. During full-text review, a VE study was excluded if it if did not meet pre-defined criteria (Supplement S3). Only VE estimates that compared fully vaccinated with unvaccinated persons were included; we excluded estimates that included partially vaccinated persons. In addition, U.S. Food and Drug Administration and European Medicines Agency websites were hand-searched for manufacturers' applications for approval of additional or booster doses. Discrepancies on study inclusion were resolved by discussion among three investigators (MH, MDK, MKP).

Most COVID-19 VE studies have given results as cumulative VE after full vaccination through variable time periods of follow-up. However, cumulative VE estimates over several months can distort estimates of waning immunity, particularly if most cases occur in the earlier or later parts of the follow-up period. Therefore, we applied a second set of inclusion/exclusion criteria after

the initial search, undertaken by two investigators (MKP, MH). First, studies were included if they presented multiple VE estimates for discrete time intervals after the final dose in the primary series. Second, to allow sufficient time for potential waning to occur, studies were excluded if they did not provide at least one VE estimate 3 months after the final dose (Supplement S4). Third, we excluded studies that combined multiple vaccines in VE estimates because vaccines of differing effectiveness were often introduced at different times to varying target populations, which could lead to confounding of VE estimates at different time intervals.

An approach to disaggregate a decreasing VE due to waning immunity from that due to a newly prevalent variant over time is to compare rates of vaccine breakthrough infections by time since vaccination during a time period when a single variant is predominant. For this approach, we considered studies of breakthrough infection (i.e., infection or disease among fully vaccinated persons only) identified through the full-text review. One study was eligible for both analyses.[10] We included studies that provided risk ratios, rate ratios, or odds ratios of breakthrough infection (or provided data to calculate them) among different vaccine-recipient cohorts (i.e., people vaccinated at different times). We only included studies that identified cases during periods when Delta was the pre-dominant variant.

All studies meeting inclusion criteria for both analyses were evaluated for bias using the risk of bias 2 tool for randomized controlled trials (ROB2) or the risk of bias in non-randomized studies of interventions tool (ROBINS-I).[11,12]

Data analysis

Populations, intervention, comparators, and outcomes (PICO) are described in Supplement S5. For the analysis of VE over time, the primary outcome measure was the VE and 95% confidence intervals (CIs) at each time interval after the final dose of the primary vaccine series. We extracted adjusted VE results for each outcome (infection, symptomatic disease, and severe disease) by vaccine, age group (all ages and older persons, as defined in each study though at least 50 years old) and variant setting. We only extracted VE estimates for time intervals when a person could have full vaccination, considered as having received the complete vaccine schedule followed by enough time to develop immunological protection, as defined in the clinical trials for each vaccine (i.e., ≥7 days from the second dose for Pfizer/BioNTech-Comirnaty, and ≥14 days from the second dose for AstraZeneca-Vaxzevria and Moderna-mRNA-1273, and the first dose of Janssen-Ad26.COV2.S.) Because VE may be lower against some variants of concern (VOC) and the prevalence of VOCs in a study population could change over time[4], we evaluated VE estimates for two variant settings separately. In the first, we evaluated VE estimates over time for a single VOC, either as determined by genomic sequencing or during a period when that variant was predominant, from settings with only non-VOC variants, and from settings with non-VOCs and Alpha variant because of minimal differences in VE.[13] In the second, we evaluated settings in which there was a mixture of variants over time, including some period with non-Alpha VOCs in circulation. To show visually the duration of VE over time, we plotted VE at the median time point for each time interval separately by outcome, age group, and variant context (Supplement S6 methods for figures.) The set of VE estimates over time for each unique study-vaccine grouping are shown joined by a line.

The average change in VE over time was estimated using a linear mixed effects model for the repeated measures within each study-vaccine group (PROC MIXED; SAS 9.4; Supplement S7 detailed methods). We regressed the log of 1-VE on log of months since vaccination (to maintain a linear relationship between VE and time in months). Standard errors (SEs) of the ln(1-VE)s, derived from the 95% CIs for the VEs reported by each study, were squared to produce estimates of residual variances for inverse weighting in the linear mixed effects model. The model had a random intercept and slope over time for each study-vaccine group (i.e., each line in figures). For VE estimates of 100% where 95% CIs were not estimable, we approximated the SEs using study data and adding 0.5 cases to each group. We excluded VE estimates with 95% CIs where the lower bound was ≤0% and upper bound was 100%, as these were uninformative. Models were run for each outcome, age group and variant context combination. Because we did not observe substantial differences in the results for single versus mixed variant settings, we also estimated the change in VE combining both variant settings to increase precision around summary estimates.

For the analysis of vaccine breakthrough Delta infections, we extracted data on study design, population size, testing period, vaccines in use, age group, outcome, cases and denominator for cohorts of persons grouped by time since final dose. We calculated incidence rates or risk from case and denominator data for each vaccinated cohort. Incidence rate/risk ratios (IRR) were calculated by dividing the incidence rate/risk of each vaccinated cohort by that of a reference group. The vaccinated cohort most recently vaccinated was used as the reference group. 95% CIs for IRRs were calculated from raw study data using the Byar method for rates and the Taylor series method for risks.[14,15] Studies presenting adjusted odds ratios of breakthrough infection

with 95% CIs were also included (n=3).[16–18] Incidence rate/risk/odds ratios with 95% CIs were graphed for each vaccinated cohort.

Role of the funding source. Coalition for Epidemic Preparedness Innovations (CEPI) supports the ongoing literature review and data abstraction. They had no role in the analysis or interpretation of results.

Results.

13,744 studies were screened, and 310 underwent full text review (Figure 1). After applying two sets of inclusion/exclusion criteria, 18 studies were included in the VE analysis. Seven were peer-reviewed publications, ten were not peer-reviewed (e.g., pre-prints, MMWR), and one came from a regulatory application. Three were randomized controlled trials[19–21] and fifteen post-introduction observational studies (7 test-negative design case-control studies, 6 retrospective and 2 prospective cohort studies, Table 1).[10,22–35] Studies were conducted in the following locations: Canada (1), Finland (1), Israel (1), Qatar (1), Spain (1), Sweden (1), United Kingdom (2), United States (8), and two multi-country clinical trials. (The Canadian study included separate results for Quebec and British Columbia, so results for each province were considered separately for this review.)[31] Among included studies, three had low overall risk of bias, eight moderate risk, and seven serious risk (Supplement S8). The major domain of bias was incomplete adjustment for confounders.

Ten studies evaluated the VE over time for SARS-CoV-2 infection, among which there were 26 vaccine-specific analyses (Pfizer/BioNTech-Comirnaty (n=13), Moderna-mRNA-1273 (n=9), Janssen-Ad26.COV2.S (n=2), AstraZeneca-Vaxzevria (n=2) (Table 1).[10,22,23,26,28,31,33–35] Ten

vaccine-specific analyses took place in single variant settings (all Delta), and 16 in mixed variant settings. Eighteen vaccine-specific analyses included persons of all ages and eight among older persons. Among the 26 vaccine-specific analyses of VE for SARS-CoV-2 infection, the majority (22, 85%) showed a ≥10 percentage point drop from the peak VE and ten (38%) a ≥25 percentage point drop (Table 2). Declines in VE against infection were observed in both variant settings, in both age groups, and among all four vaccines (Figure 2a and 2b). When combining all VE evaluations of SARS-CoV-2 infection, regardless of variant type, in the meta-regression the VE decreased on average by 21·0 (95% CI 13·9-29·8) and by 20·7 (95% CI 10·2–36·6) percentage points between 1 and 6 months after the final vaccine dose among persons of all ages and older persons, respectively.

Six studies evaluated the VE over time for symptomatic COVID-19 disease, among which there were 16 vaccine-specific analyses (Pfizer/BioNTech-Comirnaty (n=6), Moderna-mRNA-1273 (n=4), Janssen-Ad26.COV2.S (n=3), AstraZeneca-Vaxzevria (n=3) (Table 1).[19–21,25,29,30] Five vaccine-specific analyses took place in single variant settings (four Delta, one non-VOCs), and 11 in mixed variant settings. Eleven vaccine-specific analysis were done among persons of all ages and five among older persons. Among the 16 vaccine-specific analyses of VE for symptomatic disease, the majority (15, 94%) showed a ≥10 percentage point drop from the peak VE and eight (50%) a ≥25 percentage point drop, all of which were in mixed variant settings (Table 2). Declines in VE against symptomatic disease were observed in both variant settings, in both age groups, and among all four vaccines (Figure 2a and 2b). Of note, the one study that showed no decline in VE was the extended follow-up of the randomized controlled trial of the Moderna-mRNA-1273 vaccine during a period of non-VOC circulation in the United States.[19] When

combining all VE evaluations of symptomatic disease, regardless of variant type, in the meta-regression the VE decreased on average by 24·9 (95% CI 13·4-41·6) and by 32·0 (95% CI 11·0–69·0) percentage points between 1 and 6 months after the final vaccine dose among persons of all ages and older persons, respectively.

Twelve studies evaluated the VE over time for severe COVID-19 disease, among which there were 36 vaccine-specific analyses (Pfizer/BioNTech-Comirnaty (n=19), Moderna-mRNA-1273 (n=10), Janssen-Ad26.COV2.S (n=4), AstraZeneca-Vaxzevria (n=3) (Table 1).[10,21,22,24,25,27–29,31–34] Thirteen vaccine-specific analyses took place in single variant settings (eleven Delta, two Alpha), and 23 in mixed variant settings. Twenty-two vaccine-specific analysis were done among persons of all ages and fourteen among older persons. Among the 36 vaccine-specific analyses of VE for severe disease, seventeen (47%) showed a >10 percentage point drop from the peak VE (Table 2). Four vaccine-specific analyses (11%) showed a >25 percentage point drop in VE; two from one study in Qatar for Pfizer/BioNTech-Comirnaty and the other two from a study in the United States for Janssen-Ad26.COV2.S.[22,29] In both studies, the >25 percentage point VE decrease was observed among both age categories in the setting of mixed variants, with very wide 95% confidence intervals for the lowest VE estimates. Seven (19%) vaccine-specific analyses (from five studies) had an absolute VE estimate against severe disease fall below 70% at a single time point in follow-up (Pfizer/BioNTech-Comirnaty n=3, Ad26.COV2.S n=4).[21,22,27,32,33] When combining all VE evaluations of severe disease, regardless of variant type, in the meta-regression the VE decreased on average by 10·0 (95% CI 6·1-15·4) and by 9·5 (95% CI 5·7–14·6) percentage points between 1 and 6 months after the final vaccine dose among persons of all ages and older persons, respectively.

In the analysis of Delta breakthrough infections, seven studies were found through the search strategy and one through hand-searching regulatory applications; one study was excluded due to combining the results of several vaccines, leaving seven studies for final inclusion (Figure 1, Table 3). One study had low overall risk of bias, two moderate risk, and four serious risk (Supplement S8). In two clinical trials, persons initially randomized to study vaccine had an increased rate of breakthrough symptomatic COVID-19 disease during July-August 2021 when Delta variant predominated compared to those who initially received placebo and "crossed over" later to receive the actual COVID-19 vaccine; 1·76 (95% CI 1·13-2·76) times higher for Pfizer/BioNTech-Comirnaty and 1·57 (95% CI 1·21-2·04) higher for Moderna-mRNA-1273 (Figure 3).[36,37] Four observational studies from Israel of Pfizer/BioNTech-Comirnaty measured incidence after June 2021 when Delta predominated.[10,16–18] All four studies found rates of breakthrough infections higher among at least one cohort of people vaccinated further back in time compared to more recently vaccinated persons, with increased rates of breakthrough infections ranging from 1·61 times (95% CI 1·45-1·79)[16] to 14·10 times (95% CI 10.68-19.01)[17]. A study from the United States found a higher rate of breakthrough infections among persons 65 years or older vaccinated further back in time for Pfizer/BioNTech-Comirnaty (IRR 1·62, 95% CI 1·51-1·73) and Moderna-mRNA-1273 vaccines (1·67, 95% CI 1·52-1·84). Two studies evaluated breakthrough severe infections; one in Israel had a maximum of 3·25 times (95% CI 1·73-6·09) increased rates of breakthrough severe infections among persons aged 60 years or older vaccinated with Pfizer/BioNTech-Comirnaty further back in time, and one in the United States had maximum of 1·38 times (95% CI 1·18-1·62) increased rate of breakthrough hospitalized infections among

persons aged 65 years and older vaccinated with Pfizer/BioNTech-Comirnaty further back in time.[38]

Discussion

We showed that the decline in VE against severe COVID-19 disease with time since vaccination was less than that for SARS-CoV-2 infection and symptomatic COVID-19 disease. In most studies, the VE against severe disease remained high (≥70%) for up to six months post-vaccination for all four vaccines evaluated (and mostly ≥80% for the two mRNA vaccines). Nonetheless, there was a drop in VE for severe disease by six months, of on average, 9·5-10·0 percentage points, including among older persons. This lesser decrease in VE for severe disease is reassuring given that prevention of severe disease and death remains the primary objective of COVID-19 vaccination. In contrast, most studies showed a notable decrease in VE by six months after vaccination for SARS-CoV-2 infection (21 percentage point decrease) and all symptomatic COVID-19 disease (25-32 percentage point decrease). The data was heterogenous, however, with some studies showing minimal decrease in VE over time, with others showing substantial decrease (i.e., ≥25 percentage points).

A decrease in the VE over time has three potential explanations – it can reflect lower VE against a new variant, true waning immunity due to loss of vaccine-induced immunological protection, or bias. We showed that VE decreased over time when restricting analysis to a single variant. This finding was reinforced by our second analysis of breakthrough infections with the Delta variant that showed higher breakthrough rates with longer times since vaccination. Together these findings suggest that the decrease in VE over time was likely not due, for the most part, to the temporal increase in prevalence of Delta variant.

Waning VE is a plausible explanation for the decrease in VE against infection and disease. The finding is consistent with immunological data showing that over time levels of most vaccine-derived antibodies, including those that neutralize virus, decline.[39,40] Yet, because the immune system forms memory cells that can be activated upon exposure to a virus and includes cellular immunity, it is not clear if this observed antibody decay results in diminished VE and if so, over what timeframe and against which outcomes. Nevertheless, further support for possible waning immunity comes from evidence showing that after giving a booster dose the VE increases compared to persons who had only received the primary series.[41,42] Moreover, it has been shown that with increasing time since full vaccination, the viral load of breakthrough infections increases, but becomes lower again soon after booster vaccination.[43] We did not see an obvious difference in the magnitude or timing of decrease in VE between persons of all ages and older people in meta-regression, although the number of studies was likely too few to make definitive conclusions. A study from the United Kingdom showed that decreases in VE seemed to occur more among clinically extremely vulnerable older persons.[25]

While waning immunity is consistent with the data, we cannot exclude that the observed decrease in VE over time was due, either partly or wholly, to biases. An underlying assumption of observational studies is that unvaccinated persons should be at the same risk of exposure to SARS-CoV-2 as vaccinated persons in the same population. At high vaccine coverage, this assumption might no longer apply, as persons remaining unvaccinated either choose to remain unvaccinated or are unable to get vaccinated for reasons that might be associated with a differential risk of COVID-19 compared to the general population.[30,44–46] While some differences can be identified and adjusted for in the analysis (e.g., age, demographic group), others might be

less obvious, harder to measure and adjust for, and lead to underestimation of true VE over time (e.g., clinically extremely vulnerable status).[25] The expected bias based on the magnitude and direction of the differential risk of COVID-19 among unvaccinated persons demonstrates that confounding is more significant when the true VE is not as high (supplement S9); this implies that confounding by risk among the unvaccinated group is accentuated when the vaccine has lower initial efficacy and when the true VE has become lower over time.

Several other potential biases in assessing the duration of VE over time can occur (Table 4). Some important biases that could result in an overestimation of decreases in VE over time are the following: the earliest vaccinees are at sustained increased risk of infection compared to later vaccinees; vaccinated people change behavior and testing frequency over time increasing the likelihood of being infected or being detected as infected, particularly with increased mobility for those who can demonstrate vaccination status; and unvaccinated persons have increased infection-derived immunity leading to spurious interpretations of reductions in VE as waning protection.[47] Because most of these biases are unmeasured, we cannot definitely determine which ones most affected the studies included in this analysis.

Our systematic review had several other potential limitations. First, given the rapid pace and multiple pre-print publishing options for COVID-19-related content, it is possible that additional studies on vaccine duration of protection were not captured by our search strategy, and new studies will become available after our cut-off date. Second, many pre-print studies included in this analysis could have the data change in the eventual publication. Third, insufficient studies met our inclusion criteria to allow for meaningful comparisons between different vaccine platforms. Fourth, a limited number of vaccines were evaluated and from few geographic

settings, which might not be representative of other settings with different epidemiological conditions in which duration of vaccine protection might differ (e.g., more or less prior infection). Fifth, few studies evaluated VE separately in younger persons; the three studies that did so showed similar patterns of decrease in VE over time to that seen in adults of all ages and older persons (Supplement S10). Sixth, no heterologous schedules were evaluated. Seventh, all included studies were pre-Omicron. Lastly, we based our calculations on published or derived estimates of VE and their standard errors rather than original person-level event data. One manifestation of this limitation is the necessity to introduce small adjustments to VE estimates of 100% in order to include them in our model for the log-transformed relative risk estimates. The potential bias in the summary VE estimates is small because there were only three VE estimates of 100%, and two had wide confidence intervals which down-weights their contribution in the regression model.

Further follow-up of the VE against severe disease, the outcome which drives most COVID-19 policy decisions, for all vaccines beyond six months is needed to clarify how much more waning of protection might occur with longer duration since full vaccination.[48] Continuing to produce reliable and vaccine-specific VE estimates over extended periods of time after vaccination against multiple outcomes, and in the setting of emerging variants against which VE might be lower, such as Omicron, is critical for COVID-19 vaccine policy and decision-making bodies.[49] Policy makers considering the use and timing of booster doses should integrate vaccine- and outcome-specific evidence of decreasing VE with other considerations, such as vaccine coverage and supply, prioritization relative to primary series vaccination, programmatic issues, and local COVID-19 epidemiology.

Contributors.

Conceptualization -- DF, MH, MDK, MP

Data curation -- MH

Data access -- LAR, NA, YG, AH have published data that contributed to this analysis. All data included is in the public domain.

Literature search and data collection - MH, MP

Formal analysis – MDK

Funding acquisition -- Not applicable

Methodology -- MDK, SZ

Project administration -- MH

Resources -- Not applicable

Supervision -- DF

Visualization-- DF, MH, MDK, MP

Writing original draft-- DF

Data Interpretation, Writing Review and Editing—All authors

Declaration of interest.

MMH reports research grants from World Health Organization (WHO, Coalition for Epidemic Preparedness Innovations (CEPI), Asian Development Bank (ADB), Bill & Melinda Gates Foundation (BMGF), and Pfizer (all paid to the institution). RA reports a contract from the United States Centers for

Disease Control and Prevention, a grant from the Chile Ministry of Science, and consulting fees from Mayo Clinic and Chile Ministry of Health. YG reports research grants from the United States-Israel Binational Science Foundation (BSF) and Israel Science Foundation. MJG reports research grants from South African Medical Research Council and BMGF (all paid to the institution) and participation on a data safety monitoring board for a study on the effectiveness of COVID-19 vaccination against SARS-CoV-2-associated hospitalization and death. AH reports research grants from United States-Israel BSF. KLO serves as the Secretariat for the WHO Strategic Advisory Group of Experts on Immunization. MDK reports research grants from WHO, CEPI, ADB, and Pfizer (all paid to the institution) and consultancy fees from Merck.

Data sharing

All data included were derived from publicly available documents cited in the references. Extracted data are available upon request to the corresponding author.

Acknowledgments

We want to acknowledge the important contribution of the following individuals in being able to undertake this analysis: Anurima Baidya, Karoline Walter, Siqi Li, Hanane Issa, Parisa ShamaeiZadeh, and Marely Jurgensmeyer. We thank Dr. Dan-Yu Lin from University of North Carolina, Chapel Hill, USA for providing exact VE estimates and confidence intervals for his study.

References.

1 World Health Organization. Guidance document: Status of COVID-19 Vaccines within WHO EUL/PQ evaluation process. 2021 https://extranet.who.int/pqweb/sites/default/files/documents/Status_COVID_VAX_15July2021.pdf.

2 World Health Organization. COVID-19 weekly epidemiological update, edition 50, 27 July 2021. World Health Organization, 2021 https://apps.who.int/iris/handle/10665/343387 (accessed Nov 7, 2021).

3 Harder T, Koch J, Vygen-Bonnet S, *et al.* Efficacy and effectiveness of COVID-19 vaccines against SARS-CoV-2 infection: interim results of a living systematic review, 1 January to 14 May 2021. *Eurosurveillance* 2021; **26**: 2100563.

4 Harder T, Külper-Schiek W, Reda S, *et al.* Effectiveness of COVID-19 vaccines against SARS-CoV-2 infection with the Delta (B.1.617.2) variant: second interim results of a living systematic review and meta-analysis, 1 January to 25 August 2021. *Eurosurveillance* 2021; **26**: 2100920.

5 Higdon MM, Wahl B, Jones CB, *et al.* A systematic review of COVID-19 vaccine efficacy and effectiveness against SARS-CoV-2 infection and disease. *medRxiv*. 2021; (published online September 25.) (preprint). https://doi.org/10.1101/2021.09.17.21263549

6 Kow CS, Hasan SS. Real-world effectiveness of BNT162b2 mRNA vaccine: a meta-analysis of large observational studies. *Inflammopharmacol* 2021; **29**: 1075–90.

7 Meggiolaro A, Schepisi MS, Nikolaidis G, Mipatrini D, Siddu A, Rezza G. Effectiveness of vaccination against symptomatic and asymptomatic SARS-CoV-2 infection: a systematic review and meta-analysis. *medRxiv*. 2021; (published online August 28.). (preprint). https://doi.org/10.1101/2021.08.25.21262529

8 Zeng B, Gao L, Zhou Q, Yu K, Sun F. Effectiveness of COVID-19 vaccines against SARS-CoV-2 variants of concern: a systematic review and meta-analysis. 2021. *medRxiv*. 2021; (published online September 26.) (preprint). https://doi.org/10.1101/2021.09.23.21264048

9 International Vaccine Access Center. Results of COVID-19 Vaccine Effectiveness & Impact Studies: An Ongoing Systematic Review, Methods. VIEW-hub. 2021; published online July 22. https://view-hub.org (accessed Dec 8, 2021).

10 Goldberg Y, Mandel M, Bar-On YM, *et al.* Waning Immunity after the BNT162b2 Vaccine in Israel. *New England Journal of Medicine* 2021; **0**: null.

11 Sterne JA, Hernán MA, Reeves BC, *et al.* ROBINS-I: a tool for assessing risk of bias in non-randomised studies of interventions. *BMJ* 2016; **355**: i4919.

12 Sterne JAC, Savović J, Page MJ, *et al.* RoB 2: a revised tool for assessing risk of bias in randomised trials. *BMJ* 2019; **366**: l4898.

13 Weekly epidemiological update on COVID-19 - 2 November 2021. https://www.who.int/publications/m/item/weekly-epidemiological-update-on-covid-19---2-november-2021 (accessed Nov 7, 2021).

14 Breslow NE, Day NE. Statistical methods in cancer research. Volume II--The design and analysis of cohort studies. *IARC Sci Publ* 1987.

15 Kleinbaum DG, Kupper LL, Morgenstern H. Epidemiologic Research: Principles and Quantitative Methods. John Wiley & Sons, 1991.

16 Kertes J, Gez SB, Saciuk Y, *et al.* Effectiveness of the mRNA BNT162b2 vaccine six months after vaccination: findings from a large Israeli HMO. *medRxiv*. 2021; (published online September 7.) (preprint). https://doi.org/10.1101/2021.09.01.21262957.

17 Israel A, Merzon E, Schäffer AA, *et al.* Elapsed time since BNT162b2 vaccine and risk of SARS-CoV-2 infection: test negative design study. *BMJ* 2021; **375**: e067873.

18 Mizrahi B, Lotan R, Kalkstein N, *et al.* Correlation of SARS-CoV-2 Breakthrough Infections to Time-from-vaccine; Preliminary Study. *medRxiv* 2021; (published online July 31.) (preprint). https://doi.org/10.1101/2021.07.29.21261317.

19 El Sahly HM, Baden LR, Essink B, *et al.* Efficacy of the mRNA-1273 SARS-CoV-2 Vaccine at Completion of Blinded Phase. *New England Journal of Medicine* 2021; **385**: 1774–85.

20 Thomas SJ, Moreira ED, Kitchin N, *et al.* Six Month Safety and Efficacy of the BNT162b2 mRNA COVID-19 Vaccine. *medRxiv* 2021; (published online July 28): https://doi.org/10.1101/2021.07.28.21261159.

21 Janssen. Vaccines and Related Biological Products Advisory Committee October 14-15, 2021 Meeting Briefing Document - FDA (Janssen) | FDA. 2021; published online Oct 15. https://www.fda.gov/media/153037/download (accessed Nov 7, 2021).

22 Chemaitelly H, Tang P, Hasan MR, *et al.* Waning of BNT162b2 Vaccine Protection against SARS-CoV-2 Infection in Qatar. *New England Journal of Medicine* 2021; **0**: null.

23 Martínez-Baz I, Trobajo-Sanmartín C, Miqueleiz A, *et al.* Product-specific COVID-19 vaccine effectiveness against secondary infection in close contacts, Navarre, Spain, April to August 2021. *Eurosurveillance* 2021; **26**: 2100894.

24 Thompson MG, Stenehjem E, Grannis S, *et al.* Effectiveness of Covid-19 Vaccines in Ambulatory and Inpatient Care Settings. *New England Journal of Medicine* 2021; **385**: 1355–71.

25 Andrews N, Tessier E, Stowe J, *et al.* Vaccine effectiveness and duration of protection of Comirnaty, Vaxzevria and Spikevax against mild and severe COVID-19 in the UK. *medRxiv* 2021; (published online October 6.) (preprint). https://doi.org/10.1101/2021.09.15.21263583.

26 Bruxvoort KJ, Sy LS, Qian L, *et al.* Effectiveness of mRNA-1273 against Delta, Mu, and other emerging variants. *medRxiv* 2021; (published online October 1.) (preprint). https://doi.org/10.1101/2021.09.29.21264199.

27 Self WH. Comparative Effectiveness of Moderna, Pfizer-BioNTech, and Janssen (Johnson & Johnson) Vaccines in Preventing COVID-19 Hospitalizations Among Adults Without Immunocompromising Conditions — United States, March–August 2021. *MMWR Morb Mortal Wkly Rep* 2021; **70**. DOI:10.15585/mmwr.mm7038e1.

28 Tartof SY, Slezak JM, Fischer H, *et al.* Effectiveness of mRNA BNT162b2 COVID-19 vaccine up to 6 months in a large integrated health system in the USA: a retrospective cohort study. *The Lancet* 2021; **398**: 1407–16.

29 Lin D-Y, Gu Y, Wheeler B, *et al.* Effectiveness of Covid-19 Vaccines in the United States Over 9 Months: Surveillance Data from the State of North Carolina. *medRxiv* 2021; (published online October 26.) (preprint). https://doi.org/10.1101/2021.10.25.21265304.

30 Nordström P, Ballin M, Nordström A. Effectiveness of Covid-19 Vaccination Against Risk of Symptomatic Infection, Hospitalization, and Death Up to 9 Months: A Swedish Total-Population Cohort Study. *SSRN* 2021; (published online October 25.) (preprint). http://dx.doi.org/10.2139/ssrn.3949410.

31 Skowronski DM, Setayeshgar S, Febriani Y, *et al.* Two-dose SARS-CoV-2 vaccine effectiveness with mixed schedules and extended dosing intervals: test-negative design studies from British Columbia and Quebec, Canada. *medRxiv* 2021; (published online October 26.) (preprint). https://doi.org/10.1101/2021.10.26.21265397.

32 Tenforde MW. Sustained Effectiveness of Pfizer-BioNTech and Moderna Vaccines Against COVID-19 Associated Hospitalizations Among Adults — United States, March–July 2021. *MMWR Morb Mortal Wkly Rep* 2021; **70**. DOI:10.15585/mmwr.mm7034e2.

33 Irizarry RA, Robles-Fontán MM, Nieves EG, Cardona-Gerena I. Time-Varying Effectiveness of Three COVID-19 Vaccines in Puerto Rico. *SSRN* 2021; (published online October 25.) (preprint). http://dx.doi.org/10.2139/ssrn.3957118.

34 Poukka E, Baum U, Palmu AA, *et al.* Cohort study of Covid-19 vaccine effectiveness among healthcare workers in Finland, December 2020 - October 2021. *medRxiv* 2021; (published online November 8.) (preprint). https://doi.org/10.1101/2021.11.03.21265791.

35 Hall V, Foulkes S, Insalata F, *et al.* Effectiveness and durability of protection against future SARS-CoV-2 infection conferred by COVID-19 vaccination and previous infection; findings from the UK SIREN prospective cohort study of healthcare workers March 2020 to September 2021. *medRxiv* 2021; (published online December 1.) (preprint). https://doi.org/10.1101/2021.11.29.21267006.

36 Baden LR, Sahly HME, Essink B, *et al.* Covid-19 in the Phase 3 Trial of mRNA-1273 During the Delta-variant Surge. *medRxiv* 2021; (published online September 22.) (preprint). https://doi.org/10.1101/2021.09.17.21263624.

37 Pfizer Inc. BNT162b2 [Comirnaty (COVID-19 Vaccine, mRNA)] Evaluation of a Booster Dose (Third Dose) Vaccines and Related Biological Products Advisory Committee Briefing Document. Food and Drug Administration, 2021 https://www.fda.gov/media/152161/download (accessed Nov 7, 2021).

38 Rosenberg ES, Dorabawila V, Easton D, *et al.* Covid-19 Vaccine Effectiveness in New York State. *New England Journal of Medicine* 2021; **0**: null.

39 Dolgin E. COVID vaccine immunity is waning — how much does that matter? *Nature* 2021; **597**: 606–7.

40 Hamady A, Lee J, Loboda ZA. Waning antibody responses in COVID-19: what can we learn from the analysis of other coronaviruses? *Infection* 2021; published online July 29. https://doi.org.10.1007/s15010-021-01664-z.

41 Bar-On YM, Goldberg Y, Mandel M, *et al.* Protection of BNT162b2 Vaccine Booster against Covid-19 in Israel. *New England Journal of Medicine* 2021; **385**: 1393–400.

42 Barda N, Dagan N, Cohen C, *et al.* Effectiveness of a third dose of the BNT162b2 mRNA COVID-19 vaccine for preventing severe outcomes in Israel: an observational study. *The Lancet* 2021; **398**: 2093-100. https://doi.org/10.1016/S0140-6736(21)02249-2.

43 Levine-Tiefenbrun M, Yelin I, Alapi H, *et al.* Viral loads of Delta-variant SARS-CoV-2 breakthrough infections after vaccination and booster with BNT162b2. *Nat Med* 2021. https://doi.org/10.1038/s41591-021-01575-4.

44 Nunes B, Rodrigues AP, Kislaya I, *et al.* mRNA vaccine effectiveness against COVID-19-related hospitalisations and deaths in older adults: a cohort study based on data linkage of national health registries in Portugal, February to August 2021. *Eurosurveillance* 2021; **26**: 2100833.

45 Jara A, Undurraga EA, González C, *et al.* Effectiveness of an Inactivated SARS-CoV-2 Vaccine in Chile. *New England Journal of Medicine* 2021; **385**: 875–84.

46 Thompson MG, Burgess JL, Naleway AL, *et al.* Prevention and Attenuation of Covid-19 with the BNT162b2 and mRNA-1273 Vaccines. *New England Journal of Medicine* 2021; **385**: 320–9.

47 Kahn R, Schrag S, Verani J, Lipsitch M. Identifying and alleviating bias due to differential depletion of susceptible people in post-marketing evaluations of COVID-19 vaccine. *medRxiv* 2021; (published online July 19.) (preprint). https://doi.org/10.1101/2021.07.15.21260595.

48 Khoury DS, Cromer D, Reynaldi A, *et al.* Neutralizing antibody levels are highly predictive of immune protection from symptomatic SARS-CoV-2 infection. *Nat Med* 2021; **27**: 1205–11.

49 Strategic Group of Experts on Immunization. Interim statement on booster doses for COVID-19 vaccination. 2021; published online Oct 4. https://www.who.int/news/item/04-10-2021-interim-statement-on-booster-doses-for-covid-19-vaccination (accessed Nov 7, 2021).

Figure legends.

Figure 1. Study selection

Figure 2. Duration of Vaccine Effectiveness for (a) Single Variant or non-VOC settings or ((b) Mixed Variant settings

Footnote: The lower bound of 95% confidence intervals when VE=100% were undefined in manuscripts (n=1 in panel a and n=2 in panel b), and are shown here approximated (see methods in Supplementary materials S7)

Figure 3. Rate ratios of COVID-19 Breakthrough cases by time of vaccination
